# The time domain numerical method of three-dimensional conductors including radiation with lumped parameter circuit

**DOI:** 10.1038/s41598-021-83916-4

**Published:** 2021-02-25

**Authors:** Souma Jinno, Shuji Kitora, Hiroshi Toki, Masayuki Abe

**Affiliations:** Graduate School of Engineering Science, Osaka University, Toyonaka, Osaka 560-8531 Japan

**Keywords:** Electrical and electronic engineering, Electronics, photonics and device physics

## Abstract

We formulate a numerical method on the transmission and radiation theory of three-dimensional conductors starting from the Maxwell equations in the time domain. We include the delay effect in the integral equations for the scalar and vector potentials rigorously, which is vital to obtain numerically stable solutions for transmission and radiation phenomena in conductors. We provide a formalism to connect the conductors to any passive lumped-parameter circuits. We show one example of numerical calculations, demonstrating that the new formalism provides stable solutions to the transmission and radiation phenomena.

## Introduction

In the circuit design with a desire to reduce wiring widths and gaps, the shape and size of the circuit conductor significantly influence the electromagnetic performance as the signal frequency increases^1–3^. Non-uniform current distribution caused by the proximity effect and propagation in a three-dimensional conductor creates the common-mode current^4,5^.

Furthermore, the time variation of potential becomes a source of ground bounce and simultaneous switching noise^6–8^. These electromagnetic effects due to circuit geometry create various noise phenomena that can lead to miss-design of circuit performance and the generation of unexpected electromagnetic noise. For required circuit performance, the circuit design in various fields requires a robust numerical method for rigorous full-wave analysis of three-dimensional circuit structures.

The Finite-Difference Time-Domain (FDTD) method, which directly solves the Maxwell equations for electromagnetic (EM) fields in time and space, is one of the most frequently used full-wave analysis tools^9–11^. Although the FDTD method with the EM fields is suitable for analyzing the electromagnetic field caused by radiation from a circuit, it cannot precisely treat the signals flowing in the conductors connected with the lumped-parameter circuit, where potential and current are used for the time development. We have to develop a robust algorithm to treat lumped-parameter circuits connected to conductors with any shape.

There are several papers to conduct full-wave analysis, which start from the Maxwell equations in one-dimensional transmission line systems using the integral-differential equations in terms of potential and current used in the circuit theory^12–16^. There are some differences among these studies, but essentially all works emphasize that the integral-differential equations include the Heaviside equation. However, these studies did not discuss the difficulties in solving the coupled differential-integral equations numerically in the time domain with the delay term.

One attractive formalism was proposed in the name of the PEEC (Partial Element Equivalent Circuit) method, which can conduct a time-domain full-wave analysis in a three-dimensional circuit system by considering the delay time^17^. However, even in the PEEC formalism, the divergence in the numerical calculation with delay time has been a long-standing problem^18–21^. One possible cause of the numerical problem is the approximate treatment of the delay time in the Galerkin formalism with the pulse function. The delay time is approximated with a location-independent constant within finite volumes by using the center-to-center approximation^22^.

Recently, our group has successfully performed numerical calculations in the time domain, including delay times in a one-dimensional two-wire circuit system^23,24^. However, for the analysis of various applications, it is necessary to connect the external circuits, i.e., lumped-parameter circuits represented by ordinary differential equations, and extend to the three-dimensional conductor system. We note that the three-dimensional conductor can be used for a multi-layer plane conductor system by separating a three-dimensional conductor into multiple planes. We are able also to describe a system with many line conductors with multiple layer conductors.

This paper proposes a new numerical method to describe transmission and radiation phenomena in a three-dimensional conductor connected with a lumped-parameter circuit. We simultaneously discretize the space and time to rigorously treat the delay time, which depends on space in the integral equations of scalar and vector potentials. The delay difference equations are derived by discretizing Ohm’s law and continuity equation using the leap-frog method. We also made it possible to connect passive lumped-parameter circuits at the boundary^25,26^. With this new numerical method we demonstrate a numerically stable solution.

This paper is organized as follows. "Simultaneous discretization in space and time of delay-integral and partial-differential equations" section describes a simultaneous discretization method in space and time for the numerical computation of the delay integral and partial differential equations in a three-dimensional conductor. "Boundary conditions of a three-dimensional conductor with a lumped-parameter circuit" section deals with the treatment of boundary conditions and the method to connect with lumped-parameter circuits. In "Effect of the delay time in numerical results" section, we compare the results of calculations considering the treatment of delay time. “Summary” section is devoted to the summary of this paper. We add a supplemental paper to calculate the delay local coefficients of potential and inductance.

## Simultaneous discretization in space and time of delay-integral and partial-differential equations

In this paper, the potential *U*, the charge density *q*, the vector potential ${\varvec{A}}$, and the current density ${\varvec{j}}$ are used as variables to describe electromagnetic phenomena in a three-dimensional conductor. We use the integral equations for the scalar and vector potentials named as retarded potentials obtained from the Maxwell equations in the Lorenz gauge^12–16^.

1 $$\begin{aligned} U(x,y,z,t)& = \frac{1}{4\pi \varepsilon } \int _{V} \frac{ q (x',y',z', t-R({\varvec{x}}-{\varvec{x}'})/v) }{R({\varvec{x}} - {\varvec{x}'}) } {\mathrm{d}}x' {\mathrm{d}}y' {\mathrm{d}}z' ~, \end{aligned}$$

2 $$\begin{aligned} {\varvec{A}}(x,y,z,t)& = \frac{\mu }{4\pi } \int _{V} \frac{ {\varvec{j}} (x',y',z', t-R({\varvec{x}} - {\varvec{x}'})/v) }{R({\varvec{x}}-{\varvec{x}'}) } {\mathrm{d}}x' {\mathrm{d}}y' {\mathrm{d}}z' ~, \end{aligned}$$

where the distance between the two spacial positions is given as $R({\varvec{x}}-{\varvec{x}'})=\sqrt{(x'-x)^2+(y'-y)^2+(z'-z)^2}$. For stable numerical solutions, it is essential to treat the distance $R({\varvec{x}}-{\varvec{x}'})$ in the denominator and the delay time as rigorously as possible. Here, $\varepsilon$ is the permittivity and $\mu$ the permeability defined by materials surrounding conductors. The use of the Lorenz gauge over other gauges as the Coulomb gauge may have an advantage for the description of potential and current in conductors^22^.

Besides, we use the continuity equation as the charge conservation for the charge and current density in conductor. We use the Ohm’s law, ${\varvec{E}}=\rho {\varvec{j}}$, represented by partial differential equations using the potentials and current density.

3 $$\begin{aligned} \frac{ \partial q(x,y,z, t) }{ \partial t } + {\varvec{\nabla }} \cdot {\varvec{j}}(x,y,z,t)& = 0 ~, \end{aligned}$$

4 $$\begin{aligned} -{\varvec{\nabla }} U(x,y,z,t) - \frac{ \partial {\varvec{A}}(x,y,z,t) }{ \partial t }& = \rho {\varvec{j}}(x,y,z,t)~. \end{aligned}$$

Here, $\rho$ is the resistivity of the conductor. These four equations for the four quantities, $U, {\varvec{A}}, q, {\varvec{j}}$, are the fundamental equations to describe the transmission and radiation phenomena^16^. They are highly coupled equations, and we ought to develop a reliable algorithm for stable numerical solutions with suitable initial and boundary conditions. Here, the phenomenological Ohm’s law represents the effect of the electromagnetic field on the particle motion with charged particles in conductors. A microscopic derivation of the Ohm’s law from the many body quantum theory has been performed in the linear response theory^27^.

We start formulation of the numerical method for the stable solution of the above four coupled equations. First, we discretize the integral equations with the delay terms shown in Eqs. (1) and (2). Both the charge and the current exist in a three-dimensional conductor with finite volume. We use the following pulse functions to expand the charge density:

5 $$\begin{aligned} f_{j}(x)& = \left\{ \begin{array}{lll} 1 &{}\left(j-{\frac{1}{2}}\right) \Delta x \le x < \left(j+{\frac{1}{2}}\right)\Delta x , \quad (j=1,2,\cdots N_{x}) \\ 0 &{}{\mathrm{otherwise}} \end{array} \right. \end{aligned}$$

6 $$\begin{aligned} f_{k}(y)& = \left\{ \begin{array}{lll} 1 &{}\left(k-{\frac{1}{2}}\right)\Delta y \le y < \left(k+{\frac{1}{2}}\right)\Delta y, \quad (k=1,2,\cdots N_{y}) \\ 0 &{}{\mathrm{otherwise}} \end{array} \right. \end{aligned}$$

7 $$\begin{aligned} f_{l}(z)& = \left\{ \begin{array}{lll} 1 &{}\left(l-{\frac{1}{2}}\right)\Delta z \le z < \left(l+{\frac{1}{2}} \right)\Delta z, \quad (l=1,2,\cdots N_{z})\\ 0 &{}{\mathrm{otherwise}} \end{array} \right. \end{aligned}$$

8 $$\begin{aligned} g^{m}(t)& = \left\{ \begin{array}{lll} 1 &{}(m-1)\Delta t < t \le m \Delta t, \qquad \quad (m=1,2,\cdots N_{t})\\ 0 &{}{\mathrm{otherwise}} \end{array} \right. \end{aligned}$$

These pulse functions $f'$s introduce small volumes in the conductor with the size of $\Delta x \Delta y \Delta z$.

The following pulse functions are used for the current density $j_\alpha$ with $\alpha =x,y,z$:

9 $$\begin{aligned} f_{j^{-}}(x)& = \left\{ \begin{array}{lll} 1 &{}{\mathrm{for}}~~\left( j - {\frac{1}{2}}- {\frac{1}{2}}\delta (\alpha , x) \right) \Delta x \le x < \left( j+{\frac{1}{2}}-{\frac{1}{2}}\delta (\alpha , x) \right) \Delta x \\ 0 &{}{\mathrm{otherwise}}~~~~~~~~~~(j=1,2\cdots N_{x}+1) \end{array} \right. \end{aligned}$$

10 $$\begin{aligned} f_{k^{-}}(y)& = \left\{ \begin{array}{lll} 1 &{}{\mathrm{for}}~~\left( k - {\frac{1}{2}}- {\frac{1}{2}}\delta (\alpha , y)\right) \Delta y \le y < \left( k + {\frac{1}{2}}- {\frac{1}{2}}\delta (\alpha , y)\right) \Delta y \\ 0 &{}{\mathrm{otherwise}}~~~~~~~~~~ (k=1,2\cdots N_{y}+1) \end{array} \right. \end{aligned}$$

11 $$\begin{aligned} f_{l^{-}}(z)& = \left\{ \begin{array}{lll} 1 &{}{\mathrm{for}}~~\left( l - {\frac{1}{2}}- {\frac{1}{2}}\delta (\alpha , z)\right) \Delta z \le z < \left( l + {\frac{1}{2}}- {\frac{1}{2}}\delta (\alpha , z)\right) \Delta z \\ 0 &{}{\mathrm{otherwise}}~~~~~~~~~~(l=1,2\cdots N_{z}+1) \end{array} \right. \end{aligned}$$

12 $$\begin{aligned} g^{m+{\frac{1}{2}}}(t)& = \left\{ \begin{array}{lll} 1 &{}{\mathrm{for}}~~\left( m-{\frac{1}{2}}\right) \Delta t < t \le \left( m+{\frac{1}{2}}\right) \Delta t, \\ 0 &{}{\mathrm{otherwise}}~~~~~~~~~~(m=1,2\cdots N_{t}+1) \end{array} \right. \end{aligned}$$

Here, the current density’s pulse functions are shifted from that of the charge density by a half-integer multiplying the discrete size, whose direction depends on the current density component. The subscript in the upper right-hand of *j*, *k*, *l* indicates a half-integer deviation as $j^{\pm }=j\pm {\frac{1}{2}}\delta (\alpha , x)$, $k^{\pm }=k\pm {\frac{1}{2}}\delta (\alpha , y)$, $l^{\pm }=l \pm {\frac{1}{2}}\delta (\alpha , z)$. In the above pulse function only the minus subscript has been used, while the plus subscript will be used later. The charge and current densities can be expressed as follows by using the above pulse functions:

13 $$\begin{aligned} q(x,y,z,t)& = \sum _{j,k,l,m} q_{(j,k,l)}^{m} f_{j}(x) f_{k}(y) f_{l}(z) g^{m}(t) \end{aligned}$$

14 $$\begin{aligned} j_{\alpha }(x,y,z,t)& = \sum _{j,k,l,m} j_{\alpha (j^{-},k^{-},l^{-})}^{m+{\frac{1}{2}}} f_{j^{-}}(x) f_{k^{-}}(y) f_{l^{-}}(z) g^{m+{\frac{1}{2}}}(t) \end{aligned}$$

The unknown charge and current densities in the discretized space (*j*, *k*, *l*) and time (*m*) are denoted by $q_{(j,k,l)}^{m}$ and $j_{\alpha (j^{-},k^{-},l^{-})}^{m+1/2}$.

Using the collocation method, we take collocation points in space as the center of the rectangle, expressed by the pulse function. On the other hand, we noted that a collocation point of time should be the newest time defined by the pulse function. The following points define the collocation points for the charge density and the scalar potential:

15 $$\begin{aligned} x_{j}& = j \Delta x, \qquad (j = 1,2,\cdots N_{x}) \end{aligned}$$

16 $$\begin{aligned} y_{k}& = k \Delta y, \qquad (k = 1,2,\cdots N_{y}) \end{aligned}$$

17 $$\begin{aligned} z_{l}& = l \Delta z, \qquad (l = 1,2,\cdots N_{z}) \end{aligned}$$

18 $$\begin{aligned} t^{m}& = m \Delta t, \qquad (m = 1,2,\cdots N_{t}) \end{aligned}$$

On the other hand, the current density and the vector potential are defined at a position shifted from the charge density and scalar potential by a half-integer multiplying the discrete size.

19 $$\begin{aligned} x_{j^{-}}& = \left( j - {\frac{1}{2}}\delta (\alpha , x) \right) \Delta x \qquad (j=1,2\cdots N_{x}+1) \end{aligned}$$

20 $$\begin{aligned} y_{k^{-}}& = \left( k - {\frac{1}{2}}\delta (\alpha , y) \right) \Delta y \qquad (k=1,2\cdots N_{y}+1) \end{aligned}$$

21 $$\begin{aligned} z_{l^{-}}& = \left( l - {\frac{1}{2}}\delta (\alpha , z) \right) \Delta z \qquad (l=1,2\cdots N_{z}+1) \end{aligned}$$

22 $$\begin{aligned} t^{m+{\frac{1}{2}}}& = \left( m + {\frac{1}{2}}\right) \Delta t \qquad \qquad (m= 1,2\cdots N_{t}+1) \end{aligned}$$

The variables defined by the above collocation points are represented as follows:

23 $$\begin{aligned} U(x_{j},y_{k},z_{l},t^{m})& = U_{(j,k,l)}^{m} \end{aligned}$$

24 $$\begin{aligned} q(x_{j},y_{k},z_{l},t^{m})& = q_{(j,k,l)}^{m} \end{aligned}$$

25 $$\begin{aligned} A_{\alpha }(x_{j^{-}},y_{k^{-}},z_{l^{-}},t^{m+{\frac{1}{2}}})& = A_{\alpha (j^{-}, k^{-}, l^{-})}^{m+{\frac{1}{2}}} \end{aligned}$$

26 $$\begin{aligned} j_{\alpha }(x_{j^{-}},y_{k^{-}},z_{l^{-}},t^{m+{\frac{1}{2}}})& = j_{\alpha (j^{-}, k^{-}, l^{-})}^{m+{\frac{1}{2}}} \end{aligned}$$

**Figure 1:**
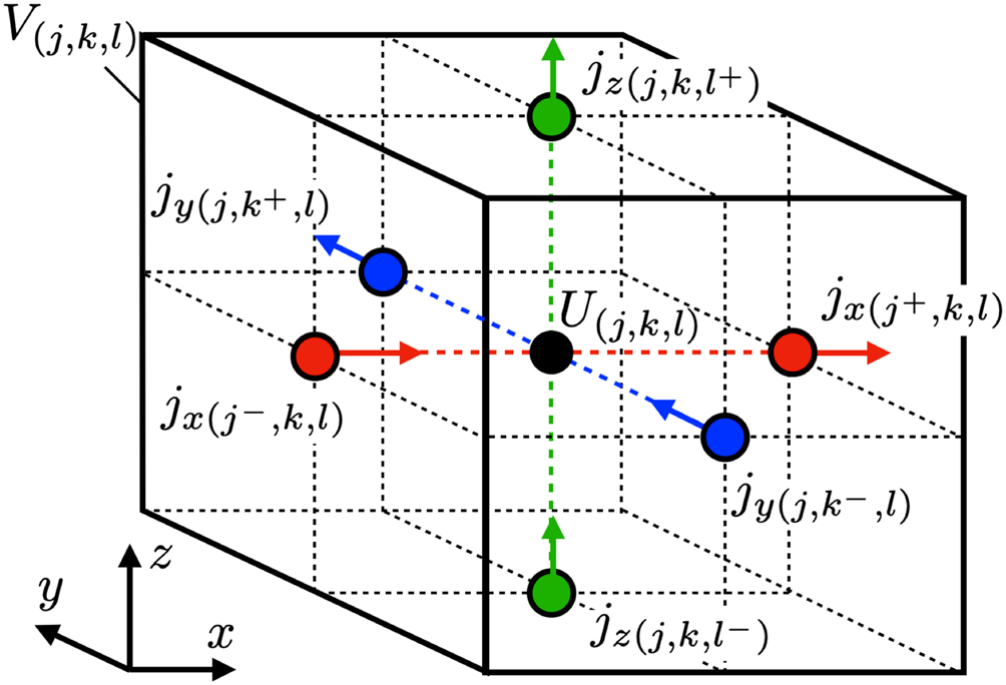
A finite volume of potential defined by the pulse functions $f_{j}(x), f_{k}(y)$ and $f_{l}(z)$ in Eqs. (5), (6) and (7) is expressed by solid line cube. The center black point denotes the potential at the collocation point. Red points represent current densities in *x*-direction, blue *y*-direction, and green *z*-direction. Collocation points of current density in *x*-, *y*- and *z*-direction are deviated by a half-integer multiple of the step size from the potential to use the leap-frog method. The deviations are expressed as $j^{\pm }=j\pm {\frac{1}{2}}\delta (\alpha , x)$, $k^{\pm }=k\pm {\frac{1}{2}}\delta (\alpha , y)$, $l^{\pm }=l \pm {\frac{1}{2}}\delta (\alpha , z)$.

**Figure 2:**
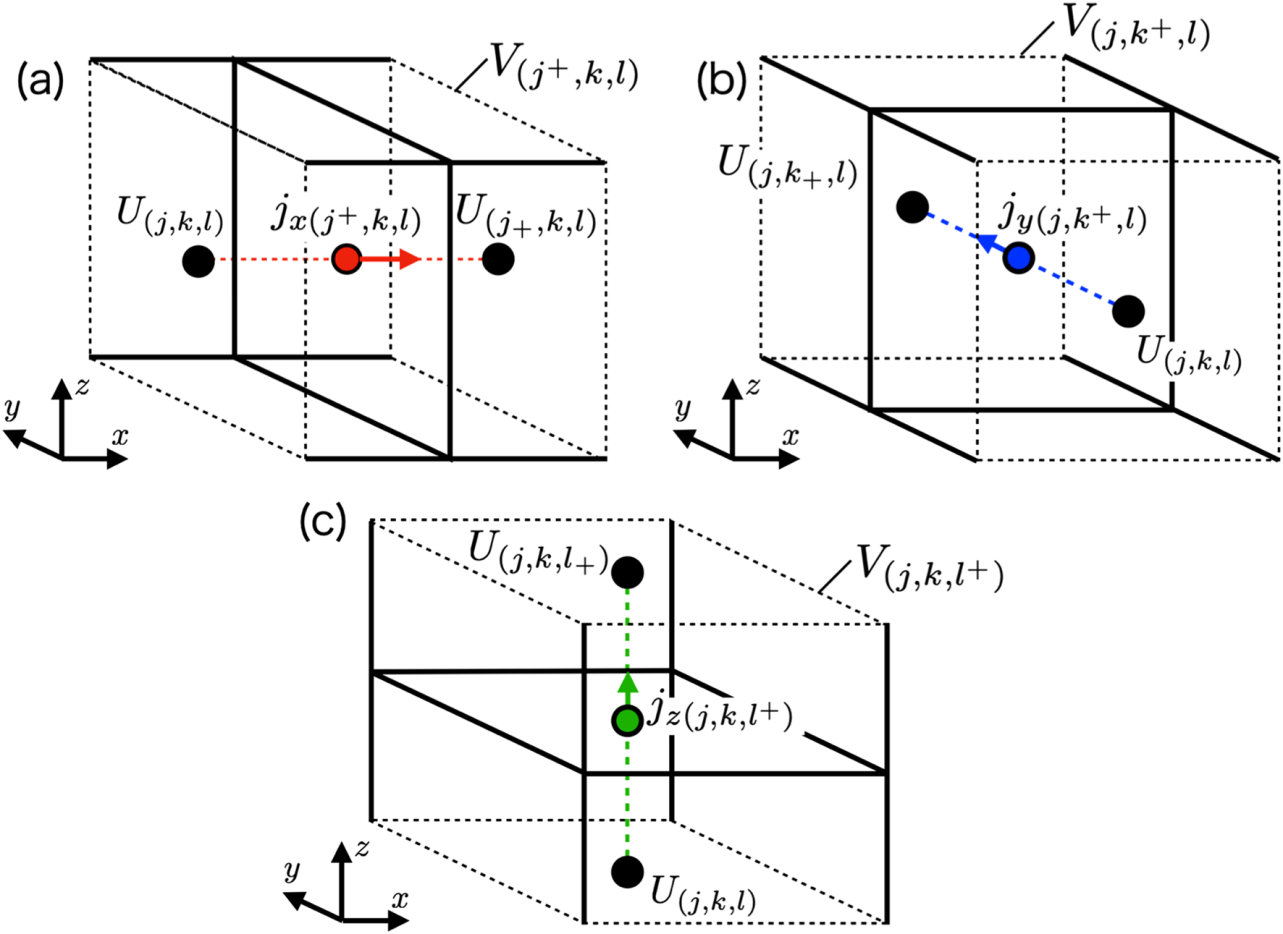
A finite volume of current densities in (**a**) *x*-, (**b**) *y*- and (**c**) *z*-direction defined by the pulse functions $f_{j^+}(x), f_{k^+}(y)$ and $f_{l^+}(z)$ in Eqs. (9), (10) and (11) is expressed by dashed line cube. Collocation points of potential are deviated by a half-integer multiple of the step size from the current density according to the direction. The deviations are expressed as $j_{\pm }=j\pm \delta (\alpha , x)$, $k_{\pm }=k\pm \delta (\alpha , y)$, $l_{\pm }=l \pm \delta (\alpha , z)$.

Figures 1 and 2 show the positional relationship between the potential and the current density defined at the collocation point in Eqs. (23) and (26).

Substituting Eqs. (13), (23) and (24) into the expression (1), we get the following expression:

27 $$\begin{aligned} U_{(j,k,l)}^{m}& = \frac{1}{4\pi \varepsilon } \int _{V}\sum _{j',k',l',m'} q _{(j',k',l')}^{m'} \nonumber \\& \quad \times \frac{ f_{j'}(x') f_{k'}(y') f_{l'}(z') g^{m'}(t^{m}-t_{(j,k,l)}(x', y', z')) }{ R(x'-x_{j}, y'-y_{k}, z'-z_{l}) } {\mathrm{d}}x' {\mathrm{d}}y' {\mathrm{d}}z' \nonumber \\& = \sum _{j',k',l',m'} q _{(j',k',l')}^{m'} \frac{1}{4\pi \varepsilon } \int _{V_{(j',k',l')}} \frac{ g^{m'}(t^{m}-t_{(j,k,l)}(x', y', z')) }{ R(x'-x_{j}, y'-y_{k}, z'-z_{l}) } {\mathrm{d}}x' {\mathrm{d}}y' {\mathrm{d}}z' ~. \end{aligned}$$

The small volume $V_{(j',k',l')}$ is obtained by the pulse function $f'$s in the *x*, *y* and *z* directions. We further point out that it is important to introduce $t_{(j,k,l)}(x', y', z')$, which is the delay time to propagate between the points $(x_{j}, y_{j},z_{k})$ and $(x',y',z')$, which are to be integrated out in the spatial integration over $x', y', z'$.

28 $$\begin{aligned} t_{(j,k,l)}(x', y', z') = \frac{ R(x'-x_{j}, y'-y_{k}, z'-z_{l}) }{ v }~, \end{aligned}$$

where *v* is the velocity of the transmission of the signal, $v=1/\sqrt{\varepsilon \mu }$.

To emphasis the delay, we use an integer *n* for the delay time from the discretized time *m*. Therefore, we rewrite $m'=m-n$ and introduce the following pulse function:

29 $$\begin{aligned} g^{n}(t)& = \left\{ \begin{array}{lll} 1 &{}n\Delta t \le t < (n+1)\Delta t, \quad (n =0,1,2,\cdots N_{d})\\ 0 &{}{\mathrm{otherwise}} \end{array} \right. \end{aligned}$$

Here, the maximum value of *n* is written as $N_{d}$, which expresses time to propagate for a signal to the farthest end of the conductor region.

30 $$\begin{aligned} N_{d} = \left\lceil \frac{\sqrt{ (\Delta xN_{x})^2 + (\Delta y N_{y})^2 + (\Delta z N_{z})^2}}{\Delta t v} \right\rceil \end{aligned}$$

$N_{x}, N_{y}$ and $N_{z}$ are numbers of divisions in *x*, *y* and *z*-direction. Finally, we can express Eq. (27) in terms of the delay time *n*.

31 $$\begin{aligned} U_{(j,k,l)}^{m}& = \sum _{j',k',l'} \sum _{n=0}^{N_{d}} q _{(j',k',l')}^{m-n} \frac{1}{4\pi \varepsilon } \int _{V_{j',k',l'}} \frac{ g^{n}(t_{(j,k,l)}(x', y', z')) }{ R(x'-x_{j}, y'-y_{k}, z'-z_{l}) } {\mathrm{d}}x' {\mathrm{d}}y' {\mathrm{d}}z' \nonumber \\& = \sum _{j',k',l'} \sum _{n=0}^{N_{d}} q _{(j',k',l')}^{m-n} P_{(j,k,l)(j',k',l')}^{n} \end{aligned}$$

Here, $P^{n}$ is called the “delay local potential coefficient,” which changes the integral range depending on the delay time *n*.

32 $$\begin{aligned} P_{(j,k,l)(j',k',l')}^{n} = \frac{1}{4\pi \varepsilon } \int _{V_{(j',k',l')}} \frac{ g^{n}(t_{(j,k,l)}(x', y', z')) }{ R(x'-x_{j}, y'-y_{k}, z'-z_{l}) } {\mathrm{d}}x' {\mathrm{d}}y' {\mathrm{d}}z' \end{aligned}$$

**Figure 3:**
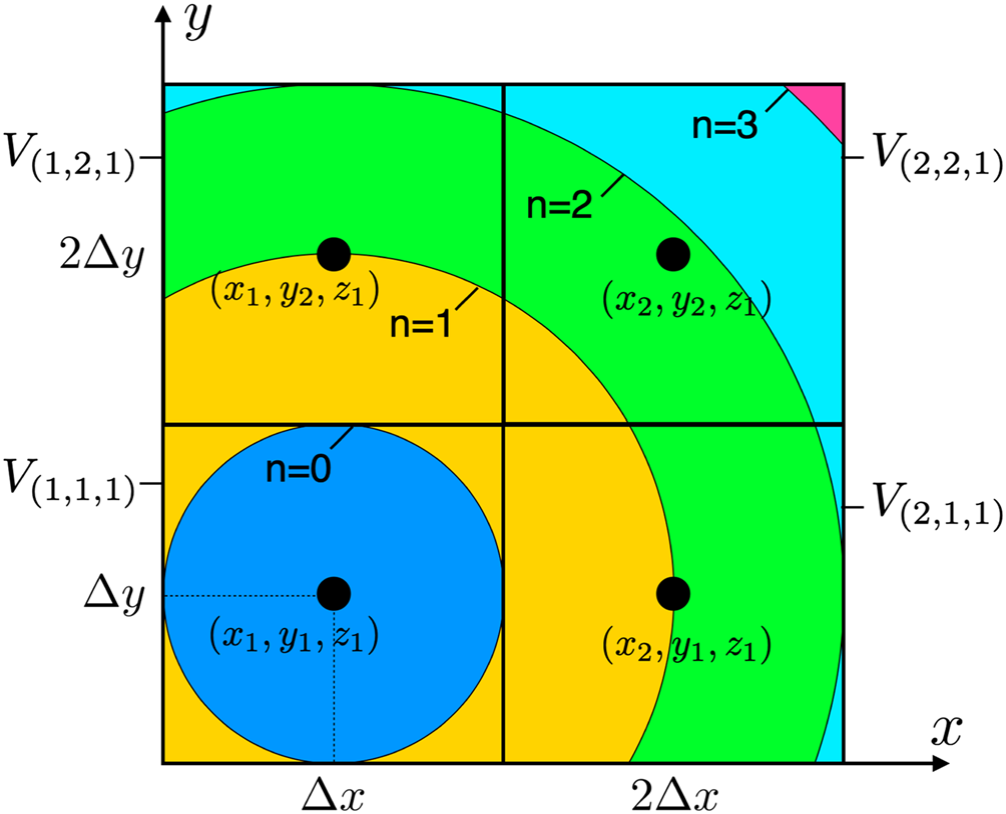
The integral range of the delay local potential coefficient $P_{(0,0,0)(j,k,l)}^{n}$ at $z=0$. The black dot represents the potential’s collocation point, and the solid square surrounding the black dot represents the finite volume of the potential for the case of $\Delta x = \Delta y$ and $\Delta t v={\frac{1}{2}}\Delta x$. The colored circle centered at the collocation point $(x_{1},y_{1},z_{1})$ has a radius of $(n+1)\Delta t v$ and represents the integral range in $(x',y',z')$ of the *n* delay effect on the colocation point $(x_{1},y_{1},z_{1})$.

The delay local potential coefficient is used to calculate the effect of charge densities in the finite volume $V_{(j',k',l')}$ on the collocation point $(x_{j},y_{k},z_{l})$, and the range of the effect is shaved in $V_{(j',k',l')}$ by the function $g^{n}(t_{(j,k,l)}(x',y',z'))$ as shown in Fig. 3. In the conventional method, one uses the center-to-center approximation, where the $x',y',z'$ coordinates in the delay time are approximated to be at the collocation point of the finite volume $V_{(j',k',l')}$ ^22^. We are then able to take out the charge density with the delay time from the integral over $x',y',z'$, and the integrand becomes simply $1/R(x'-x_{j}, y'-y_{k}, z'-z_{l})$ in the cubic $V_{(j',k',l')}$. Although the calculation of the local potential coefficient becomes simple, this center-to-center approximation leads to coupled delayed partial differential equations.

The vector potential can be also expressed in the same way using the equations (2), (14), (25), and (26):

33 $$\begin{aligned} A_{\alpha (j^{-},k^{-},l^{-})}^{m+{\frac{1}{2}}} = \sum _{j',k',l'} \sum _{n=0}^{N_{d}} j_{\alpha (j'^{-},k'^{-},l'^{-})}^{m+{\frac{1}{2}}-n} L_{\alpha (j^{-},k^{-},l^{-})(j'^{-},k'^{-},l'^{-})}^{n} \end{aligned}$$

Here, $L_{\alpha (j^{-},k^{-},l^{-})(j^{'-},k^{'-},l^{'-})}^{n}$ is the “delay local inductance”, and the integral range depends on the delay time *n*.

34 $$\begin{aligned} L_{\alpha (j^{-},k^{-},l^{-})(j'^{-},k'^{-},l'^{-})}^{n} = \frac{\mu }{4\pi } \int _{V_{\alpha (j',k',l')}} \frac{ g^{n}\left( t_{\alpha (j^{-},k^{-},l^{-})}(x', y', z') \right) }{ R(x'-x_{j^{-}}, y'-y_{k^{-}}, z'-z_{l^{-}}) } {\mathrm{d}}x' {\mathrm{d}}y' {\mathrm{d}}z' \end{aligned}$$

Next, we discretize the continuity equation (3) using the FDTD method, which is the central difference method for the differential. The discretized space and time of the charge and current deviate from each other by a half integer in both the space and time directions.

35 $$\begin{aligned} \frac{ q_{(j,k,l)}^{m+1} - q_{(j,k,l)}^{m} }{ \Delta t } + \sum _{\alpha } \frac{ j_{\alpha (j^+,k^+,l^+)}^{m+{\frac{1}{2}}} - j_{\alpha (j^-,k^-,l^-)}^{m+{\frac{1}{2}}} }{ \Delta \alpha } = 0~. \end{aligned}$$

Here, the collocation points shifted by a half-integer as $j^{\pm }=j\pm {\frac{1}{2}}\delta (\alpha , x)$, $k^{\pm }=k\pm {\frac{1}{2}}\delta (\alpha , y)$, $l^{\pm }=l \pm {\frac{1}{2}}\delta (\alpha , z)$ as the one defined before.

Similarly, we discretize Ohm’s law (4) in the $\alpha$ direction.

36 $$\begin{aligned}- \frac{ U_{(j,k,l)}^{m+1} - U_{(j_-, k_-,l_-)}^{m+1} }{ \Delta \alpha } - \frac{ A_{\alpha (j^-,k^-,l^-)}^{m+\frac{3}{2}} - A_{\alpha (j^-,k^-,l^-)}^{m+{\frac{1}{2}}} }{ \Delta t } = \rho \frac{ j_{\alpha (j^-,k^-,l^-)}^{m+\frac{3}{2}} + j_{\alpha (j^-,k^-,l^-)}^{m+\frac{1}{2}} }{ 2 }~. \end{aligned}$$

Here, we define the collocation points shifted by an integer as $j_{\pm }=j\pm \delta (\alpha , x)$, $k_{\pm }=k\pm \delta (\alpha , y)$, $l_{\pm }=l\pm \delta (\alpha , z)$. Using Eqs. (31), (33), (35) and (36), we can derive the unknown quantities, $U, q, A_\alpha$ and $j_\alpha$ at an advanced time using all the corresponding past quantities.

Furthermore, we derive the delay difference equations in terms of the potential and current used in the circuit theory. From Eqs. (31) and (35), after eliminating the charge density, the updated equation for the potential is expressed as follows:

37 $$\begin{aligned} \frac{ U^{m+1}_{(j,k,l)} - U^{m}_{(j,k,l)} }{ \Delta t }& = \sum _{j',k',l'} \sum _{n} P_{(j,k,l)(j',k',l')}^{n} \frac{ q_{(j',k',l')}^{m+1-n} - q_{(j',k',l')}^{m-n} }{ \Delta t} \end{aligned}$$

Transferring the unknown potential at an advanced time to the left-hand side, the updating equation for the potential can be expressed as follows using the current densities with the continuity equation (35):

38 $$\begin{aligned} U_{(j,k,l)}^{m+1}& = U_{(j,k,l)}^{m} - \sum _{\alpha } \sum _{j',k',l'} \sum _{n} \frac{\Delta t}{\Delta \alpha } P_{(j,k,l)(j',k',l')}^{n} \left( j_{\alpha (j^+,k^+,l^+)}^{m+{\frac{1}{2}}-n} - j_{\alpha (j^-,k^-,l^-)}^{m+{\frac{1}{2}}-n} \right) \end{aligned}$$

Similarly, by eliminating the vector potential from Eqs. (33), (36) and transposing the new current density to the left-hand side, the updated equation for the current density is expressed as follows:

39 $$\begin{aligned}&\sum _{j',k',l'} \left( \frac{\Delta \alpha }{\Delta t} L_{\alpha (j^-,k^-,l^-)(j^{'-},k^{'-},l^{'-})}^{0} + \frac{\Delta \alpha \rho }{2} \right) j_{\alpha (j^{'-},k^{'-},l^{'-})}^{m+\frac{3}{2}} \nonumber \\&\quad = \sum _{j',k',l'} \left( \frac{\Delta \alpha }{\Delta t} L_{\alpha (j^-,k^-,l^-)(j^{'-},k^{'-},l^{'-})}^{0} - \frac{\Delta \alpha \rho }{2} \right) j_{\alpha (j^{'-},k^{'-},l^{'-})}^{m+\frac{1}{2}} \nonumber \\& \qquad - \sum _{j',k',l'} \sum _{n=1}^{N} \frac{\Delta \alpha }{\Delta t} L_{(j^-,k^-,l^-)(j'^-,k'^-,l'^-)}^{n} \left( j_{\alpha (j'^-,k'^-,l'^-)}^{m+\frac{3}{2} - n} - j_{\alpha (j'^-,k'^-,l'^-)}^{m+{\frac{1}{2}}- n} \right) \nonumber \\& \qquad - \left( U_{(j,k,l)}^{m+1} - U_{(j_-,k_-,l_-)}^{m+1} \right) \end{aligned}$$

Here, in the case $\Delta \alpha /2 < v\Delta t$, the non-delay local mutual inductance $L^{0}$ becomes finite for neighboring cells, and simultaneous equations are necessary to be solved. On the other cases, when the neighboring non-delay local inductance is zero for $\Delta \alpha /2 \ge v\Delta t$, and the current density at each location can be solved directly:

40 $$\begin{aligned} j_{\alpha (j^{-},k^{-},l^{-})}^{m+\frac{3}{2}}& = \frac{ \left( \frac{\Delta \alpha }{\Delta t} L_{(j^{-},k^{-},l^{-})(j^{-},k^{-},l^{-})}^{0} - \frac{\Delta \alpha \rho }{2} \right) }{ \left( \frac{\Delta \alpha }{\Delta t} L_{(j^{-},k^{-},l^{-})(j^{-},k^{-},l^{-})}^{0} + \frac{\Delta \alpha \rho }{2} \right) } j_{\alpha (j^{-},k^{-},l^{-})}^{m+\frac{1}{2}} \nonumber \\&\quad - \sum _{j',k',l'} \sum _{n=1}^{N_{d}} \frac{ \frac{\Delta \alpha }{\Delta t} L_{(j^{-},k^{-},l^{-})(j^{'-},k^{'-},l^{'-})}^{n} }{ \left( \frac{\Delta \alpha }{\Delta t} L_{(j^{-},k^{-},l^{-})(j^{'-},k^{'-},l^{'-})}^{0} + \frac{\Delta \alpha \rho }{2} \right) } \left( j_{\alpha (j^{'-},k^{'-},l^{'-})}^{m+\frac{3}{2}-n} - j_{\alpha (j^{'-},k^{'-},l^{'-})}^{m-\frac{1}{2}-n} \right) \nonumber \\&\quad - \frac{ U_{(j, k, l)}^{m+1} - U_{(j_-,k_-,l_-)}^{m+1} }{ \left( \frac{\Delta \alpha }{\Delta t} L_{(j^{-},k^{-},l^{-})(j^{-},k^{-},l^{-})}^{0} + \frac{\Delta \alpha \rho }{2} \right) } \end{aligned}$$

Using Eqs. (38) and (40), we are able to obtain the most advanced potential *U* and current density $j_\alpha$ using known past potentials and current densities.

## Boundary conditions of a three-dimensional conductor with a lumped-parameter circuit

**Figure 4:**
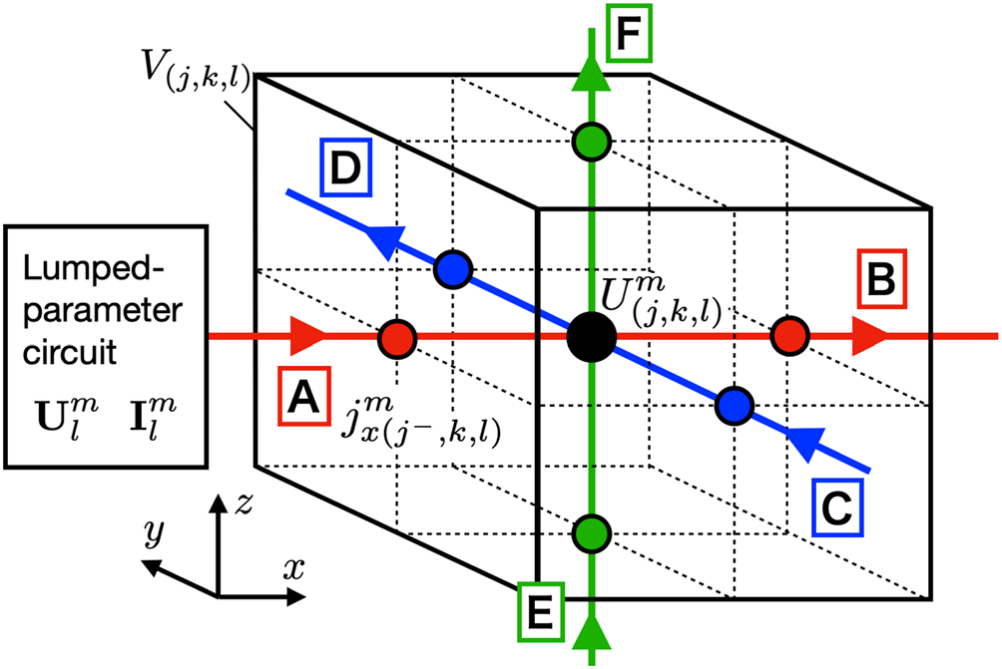
The connection between the potentials and current densities in lumped-parameter and three-dimensional circuits at the boundary is shown. The node potential of the lumped-parameter circuit $U^m_l$ and the potential of the three-dimensional circuit at the boundary $U_{(j,k,l)}^m$ are equivalent, and the branch current of the lumped-parameter circuit $I^m_l$ and the current of the three-dimensional circuit at the boundary $j_{x(j^-,k,l)}^m$ are equivalent. The direction of the current is perpendicular to the surface of the boundary. There are six different surfaces from *A* to *F* as the boundary. In the figure shown here, as an example, the lumped-parameter circuit is connected at the surface *A*.

In this section, we discuss the treatment of potentials and current densities at the boundaries between a conductor and a lumped-parameter circuit. We consider a case where the current density flows to the surface *A* of a conductor in the vertical direction through a lumped parameter circuit as shown in Fig. 4. There are other surfaces *B*, *C*, *D*, *E*, *F* in Fig. 4, where a lumped parameter circuit may be connected. We derive a boundary condition to connect the lumped-parameter circuit to the delay difference equations we have derived in “Simultaneous discretization in space and time of delay-integral and partial-differential equations” section.

We use a nodal potential and a branch current as unknown variables in the lumped-parameter circuit, considering the nodal potential to be equivalent to the potential of the conductor and the branch current equivalent to the conductor’s current at the boundary. In a previous study, based on the sparse-tableau method, we have derived an update equation in the time domain for the nodal potential ${\mathbf{U}}_{l}$ and branch current ${\mathbf{I}}_{l}$ of the lumped-parameter circuit^26^. We can summarize the branch constitutive equation (BCE), Kirchhoff’s voltage law (KVL), and Kirchhoff’s current law (KCL) in a matrix and vector form^26^.

41 $$\begin{aligned}\left( \begin{array}{ccc} {\mathbf{A}}_{l}^{T} &{}-\mathbf{Z}_{l} \\ \mathbf{0} &{}{\mathbf{A}}_{l} \end{array} \right) \left( \begin{array}{c} {\mathbf{U}}_{l}^{m+1} \\ {\mathbf{I}}_{l}^{m+1} \end{array} \right) = \left( \begin{array}{ccc} -{\epsilon }{\mathbf{A}}_{l}^{T} &{}{\delta }{} \mathbf{Z}_{l} \\ \mathbf{0} &{}{\mathbf{A}}_{l} \end{array} \right) \left( \begin{array}{c} {\mathbf{U}}_{l}^{m} \\ {\mathbf{I}}_{l}^{m} \end{array} \right) \nonumber + \left( \begin{array}{c} \mathbf{V}_{s}^{m+1} + \mathbf{V}_{s}^{m} \\ -{\mathbf{A}}_{s} {\mathbf{I}}_{s}^{m+1} \end{array} \right) \end{aligned}$$

Here, ${\mathbf{U}}_{l}$ and ${\mathbf{I}}_{l}$ represents the nodal potential and the branch current, which are unknowns in the lumped-parameter circuit. ${\varvec{A}}_{l}$ is an incidence matrix representing the node-to-branch connection relationship and the direction. Their components are 0, 1 and $-1$, where the row element corresponds to the node, and the column is the branch. For example, when the node *p* and the branch *q* are not connected, the matrix element at the *p*th row and *q*th column is 0. When the node and branch are connected, and the direction is outward from the node, the matrix element is 1, and when the direction is inward from the node, the element is – 1. $\mathbf{Z}_{l}$ is the time-domain impedance matrix, which is a diagonal matrix. The matrix element in the *q*th row and *q*th column are *R* when the branch *q* has a resistor *R*, $\Delta t/(2C)$ for a capacitor *C*, and $2L/(\Delta t)$ for an inductor *L*. Besides, $\mathbf{V}_{s}$ represents the voltage source vector, and the *q*th element has the value of the voltage source in the branch *q*. ${\mathbf{I}}_{s}$ represents the current source vector, and *q*th element has the value of the current source in the branch *q*.

The matrices $\epsilon$ and $\delta$ are diagonal matrices for the sign dependence of the electric elements in the branch, and the diagonal $\beta$th element is represented as follows:

42 $$\begin{aligned} \epsilon _{\beta \beta } = \left\{ \begin{array}{cl} -1 &{} \mathrm{for\ capacitor} \\ 1 &{} \mathrm{for\ other\ elements} \end{array} \right. ,\ \delta _{\beta \beta } = \left\{ \begin{array}{cl} -1 &{} \mathrm{for\ inductor} \\ 1 &{} \mathrm{for\ other\ elements} \end{array} \right. \end{aligned}$$

Next, we write the equation that the delay difference equation (38) satisfies at the boundary. There is no concept of space in the lumped-parameter circuit, and we identify the potential of the lumped-parameter circuit to the potential of the three-dimensional conductor at the boundary. In the example shown in Fig. 4, $U_l^m$ is set equal to $U_{(j,k,l)}^m$. On the other hand, the current density is defined at a half integer time in the delay differential equation as shown in Eq. (38). Hence, we need to introduce the current density at the boundary in the integer multiple time steps. To this end, we introduce the current density of the integer time using the time average:

43 $$\begin{aligned} j_{\alpha (j^{\pm },k^{\pm },l^{\pm })}^{m+{\frac{1}{2}}} = \frac{ j_{\alpha (j^{\pm },k^{\pm },l^{\pm })}^{m+1} + j_{\alpha (j^{\pm },k^{\pm },l^{\pm })}^{m} }{ 2 } \end{aligned}$$

We can get boundary condition, substituting Eq. (43) into (38), converting the time of the boundary current density to the integer, and transferring the unknowns to the left side.

44 $$\begin{aligned}&U^{m+1}_{(j,k,l)} - \sum _{\alpha }^{x,y,z} \sum _{j',k',l'}^{boundary} \frac{1}{2}\gamma _{\alpha (j',k',l')} \frac{\Delta t}{\Delta \alpha } P_{(j,k,l)(j',k',l')}^{0} j_{\alpha (j^{'\pm },k^{'\pm },l^{'\pm })}^{m+1} \nonumber \\&\quad = U^{m}_{(j,k,l)} + \sum _{\alpha }^{x,y,z} \sum _{j',k',l'}^{boundary} \frac{1}{2}\gamma _{\alpha (j',k',l')} \frac{\Delta t}{\Delta \alpha } P_{(j,k,l)(j',k',l')}^{0} j_{\alpha (j^{'\pm },k^{'\pm },l^{'\pm })}^{m} \nonumber \\&\qquad - \sum _{\alpha }^{x,y,z} \sum _{j',k',l'}^{boundary} \gamma _{\alpha (j',k',l')} \frac{\Delta t}{\Delta \alpha } P_{(j,k,l)(j',k',l')}^{0} j^{m+{\frac{1}{2}}}_{\alpha (j^{'\mp }, k^{'\mp }, l^{'\mp })} \nonumber \\&\qquad - \sum _{\alpha }^{x,y,z} \sum _{j',k',l'}^{no~boundary} \sum _{n}\, \frac{\Delta t}{\Delta \alpha } P_{(j,k,l)(j',k',l')}^{n} \Big ( j^{m+{\frac{1}{2}}-n}_{\alpha (j^{'+},k^{'+},l^{'+})} -j^{m+{\frac{1}{2}}-n}_{\alpha (j^{'-},k^{'-},l^{'-})} \Big ) ~. \end{aligned}$$

Here, $\gamma _{\alpha (j,k,l)}$ is a coefficient for the sign dependence of the direction of the current density, which depends on the location where the lumped-parameter circuit is connected as shown in Fig. 4 and its value is shown in Table 1. We note that the current density defined at the integer time is zero at the boundary where no lumped parameter circuit is connected, since there is no current flowing in and out through lumped-parameter circuit.

**Table 1:** Coefficients $\gamma _{x}$, $\gamma _{y}$, and $\gamma _{z}$ are depending on the lumped-parameter circuit’s connection between the nodes of potential (*j*, *k*, *l*) and current density $(j^{\pm },k^{\pm },l^{\pm })$ at the boundaries A through F shown in Fig. 4.

Position	A	B	C	D	E	F
$\gamma _{x}$	1	– 1	0	0	0	0
$\gamma _{y}$	0	0	1	– 1	0	0
$\gamma _{z}$	0	0	0	0	1	– 1

By summarizing the potential and current density at the collocation point where the lumped-parameter circuit is connected, we write Eq. (44) in the matrix form using vectors ${\mathbf{U}}_{d}$ and $\mathbf{j}_{d}$.

45 $$\begin{aligned} {\mathbf{A}}_{d}^{T} {\mathbf{U}}_{d}^{m+1} - \mathbf{Z}_{d}^{0} \mathbf{j}_{d}^{m+1} = {\mathbf{A}}_{d}^{T} {\mathbf{U}}_{d}^{m} + \mathbf{Z}_{d}^{0} \mathbf{j}_{d}^{m} - {\tilde{U}}_{d}^{m+1}~. \end{aligned}$$

Here, ${\mathbf{A}}_{d}$ is an incidence matrix representing the connection relationship between the potential ${\mathbf{U}}_{d}$ and the current $\mathbf{j}_{d}$ at the boundary. Since the direction of the current is determined by $\gamma _{\alpha }$, the matrix elements of ${\mathbf{A}}_{d}$ are 1 or 0. The rows correspond to the potential and the columns to the current density. For example, when the potential *p* and the current density *q* are connected to the same lumped-parameter circuit, the element in the *p*th row *q*th column will be 1. On the other hand, when it is not connected, it will be 0. ${\varvec{Z}}_{d}^{0}$ is a non-delay local impedance matrix, and the element in the *p*th row *q*th column is $\frac{1}{2}\gamma _{\alpha (q)}\frac{\Delta t}{\Delta \alpha }P_{pq}^{0}$ where *p* and *q* are collocation points of potential. Besides, $\mathbf{\tilde{U}}_{d}$ is a delay term vector, which is a contribution to the boundary potential and represents the third and fourth items on the right side of Eq. (44).

Finally, we use Kirchhoff’s current law to describe the connection between the lumped-parameter circuit and the three-dimensional conductor.

46 $$\begin{aligned} \left( {\gamma }_{x}{} \mathbf{S}_{x} + {\gamma }_{y}{} \mathbf{S}_{y} + {\gamma }_{z}{} \mathbf{S}_{z} \right) {\mathbf{A}}_{d} \mathbf{j}_{d}^{m+1} + {\mathbf{A}}_{l}{} {\mathbf{I}}_{l}^{m+1} = - {\mathbf{A}}_{s}{} {\mathbf{I}}_{s}^{m+1}~. \end{aligned}$$

Here, ${\gamma }_{\alpha }{\varvec{S}}_{\alpha }$ is a diagonal matrix whose components are multiplication of $\gamma _{\alpha }$ and cross-sections of finite volume in the $\alpha (=x,y,z)$ direction. The above equations (41), (45) and (46) are the boundary conditions. To solve these equations, we write a matrix expression of the same form as the expression (41).

First, the unknown variables ${\mathbf{U}}_{d}, {\mathbf{U}}_{l}, {\mathbf{I}}_{l}$ and $\mathbf{j}_{d}$ are summarized below.

47 $$\begin{aligned} {\mathbf{U}} = \left( \begin{array}{cc} {\mathbf{U}}_{d} \\ {\mathbf{U}}_{l} \end{array} \right) ,\ {\mathbf{I}} = \left( \begin{array}{cc} {\mathbf{I}}_{l} \\ \mathbf{j}_{d} \end{array} \right) ~. \end{aligned}$$

The incidence matrixes for the lumped-parameter and three-dimensional circuits are summarized below.

48 $$\begin{aligned} \mathbf {A} = \left( \begin{array}{ccc} {\mathbf{A}}_{l}&{\mathbf{A}}_{d} \end{array} \right) \end{aligned}$$

The impedance matrices of the lumped-parameter and three-dimensional circuits are summarized below.

49 $$\begin{aligned} \mathbf{Z} = \left( \begin{array}{ccc} \mathbf{Z}_{l} &{}\mathbf{0} \\ \mathbf{0} &{}\mathbf{Z}_{d} \end{array} \right) \end{aligned}$$

The lumped-parameter circuit’s voltage source and the delay term in the delay difference equation are summarized as follows.

50 $$\begin{aligned} \mathbf{E}^{m+1} = \left( \begin{array}{cc} \mathbf{V}_{s}^{m+1} + \mathbf{V}_{s}^{m} \\ {\tilde{{\mathbf{U}}}}_{d}^{m+1} \end{array} \right) \end{aligned}$$

The boundary conditions for connecting the lumped-parameter and three-dimensional circuits can be expressed as follows from the above expressions.

51 $$\begin{aligned} \left( \begin{array}{ccc} {\mathbf{A}}^{T} &{} -\mathbf{Z} \\ \mathbf{0} &{} {\gamma }_{S}{} {\mathbf{A}} \end{array} \right) \left( \begin{array}{cccccc} {\mathbf{U}}^{m+1} \\ {\mathbf{I}}^{m+1} \end{array} \right) = \left( \begin{array}{ccc} -{\epsilon }{\mathbf{A}}^{T} &{}{\delta }{} \mathbf{Z} \\ \mathbf{0} &{} \mathbf{0} \end{array} \right) \left( \begin{array}{cccccccccc} {\mathbf{U}}^{m} \\ {\mathbf{I}} ^{m} \end{array} \right) + \left( \begin{array}{cccccccccc} \mathbf{E}^{m+1} \\ -{\mathbf{A}}_{s}{\mathbf{I}}_{s}^{m+1} \end{array} \right) \end{aligned}$$

Here, $\varepsilon$ and $\delta$ are matrices for changing the sign depending on the components of the lumped-parameter and three-dimensional circuits.

52 $$\begin{aligned} \epsilon _{\beta \beta }& = \left\{ \begin{array}{cl} -1 &{} \mathrm{for\ capacitor\ and\ three\ dimensional\ circuit} ~, \\ 1 &{} \mathrm{otherwise} ~. \end{array} \right. \end{aligned}$$

53 $$\begin{aligned} \delta _{\beta \beta }& = \left\{ \begin{array}{cl} -1 &{} \mathrm{for\ inductor} ~, \\ 1 &{} \mathrm{otherwise} ~.\\ \end{array} \right. \end{aligned}$$

Furthermore, $\gamma _{S}$ is the matrix for converting the units of current density in the three-dimensional circuit to current in Kirchhoff’s current law.

54 $$\begin{aligned} {\gamma _{S}}_{\beta \beta } = \left\{ \begin{array}{cl} 1 &{} \mathrm{for\ lumped~parameter\ elements} , \\ \gamma _{x\beta \beta }S_{x\beta \beta } + \gamma _{y\beta \beta }S_{y\beta \beta } + \gamma _{z\beta \beta }S_{z\beta \beta } &{} \mathrm{for~three~ dimensional~circuit} . \end{array} \right. \end{aligned}$$

## Effect of the delay time in numerical results

In this section, we discuss the treatment of delay time for numerical calculations in the time domain. Compared to the rigorous method presented in this paper without approximation of delay time, we use the center-to-center approximation, which has been adopted in the PEEC method^17^.

55 $$\begin{aligned} t_{(j,k,l)({j'},{k'},{l'})} = \frac{ R(x_{j'}-x_j,y_{k'}-y_k,z_{l'}-z_l) }{ v }~. \end{aligned}$$

Here, $x_i, y_k, z_l$ and $x_{j'} ,y_{k'}, z_{l'}$ are the collocation points. Exploiting this approximation, we can discretize the charge and current densities in time, including the delay time, which does not influence the integral over space in the local impedance. Since we can pull out the charge and current densities from the space integral, we obtain a simplified delay local impedance:

56 $$\begin{aligned} Z_{(j,k,l)(j',k',l')}^{n} = \frac{1}{4\pi } \sqrt{\frac{\mu }{\varepsilon }} \int _{V_{(j',k',l')}} \frac{ 1 }{ R(x'-x_{j}, y'-y_{k}, z'-z_{l}) } {\mathrm{d}}x' {\mathrm{d}}y' {\mathrm{d}}z' \end{aligned}$$

Here, in the center-to-center approximation, the discretized delay time *n* is uniquely defined by the pulse function in Eq. (29) regardless of the space integral range, which is equivalent to the approximation using floor function^28^.

**Figure 5:**
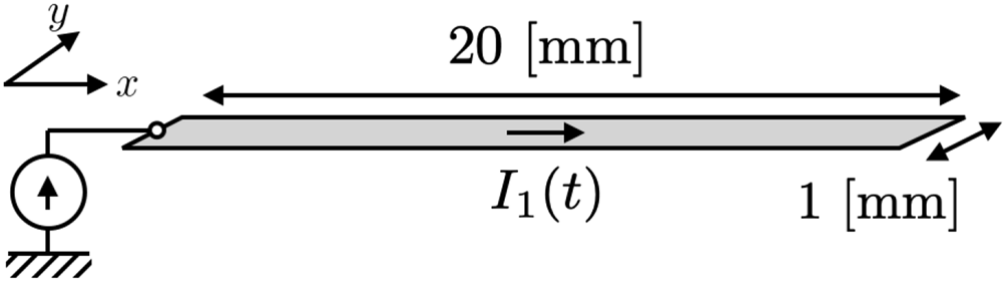
A single-plane circuit consists of a conductor plane of 1 mm width and 20 mm length. In numerical calculations, the step size in space is set to $\Delta x = \Delta y = 0.2$mm and the step size in time is set to $\Delta t v= \Delta x/2$, where *v* is the velocity of the signal. A current source is connected to the left end of the single-plane circuit, where pulse current is used as input to the boundary. The boundary was calculated numerically using the method described in “Boundary conditions of a three-dimensional conductor with a lumped-parameter circuit” section.

**Figure 6:**
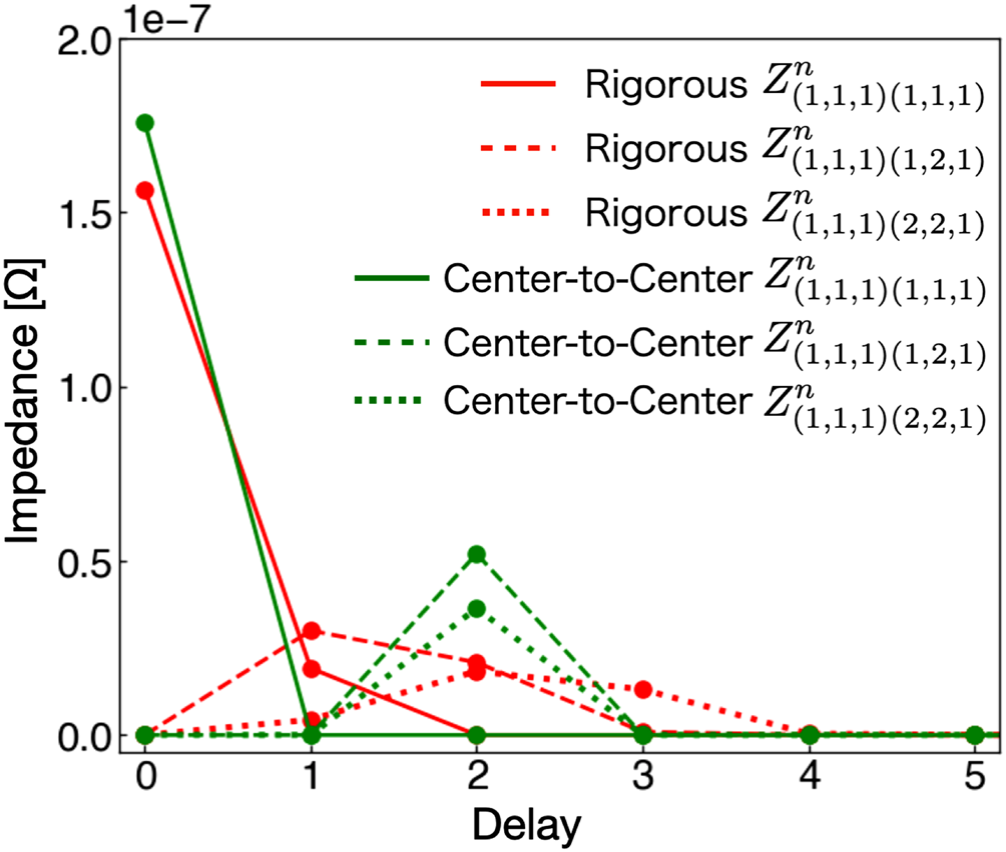
Delay local impedances between two finite volumes after discretizing the single-plane circuit as functions of discretized delay time *n*. The solid line represents the delay local self-impedance $Z^{n}_{(1,1,1)(1,1,1)}$. The dashed and dotted lines are the delay local mutual impedances $Z^{n}_{(1,1,1)(1,2,1)}$ and $Z^{n}_{(1,1,1)(2,2,1)}$ between the neighboring cells of the origin $(x_{1},y_{1},z_{1})$, respectively. The red lines denote the result of the rigorous method presented in this paper, the green lines the center-to-center approximation method. In the Supplementary Information, we explain the detailed calculation method of delay local impedance in the plane circuit.

For the demonstration of the two treatments of the delay time, we use a single-plane circuit consisting of a plane conductor as shown in Fig. 5. The step size in space is $\Delta x= \Delta y = 0.2$ mm and the step size in time is $0.5\times \Delta x/v$. Figure 6 shows the numerical results of delay local self- and mutual-impedances in this numerical condition. Here, the calculation methods of delay local impedance are described in the Supplementary Information. When we rigorously treat the delay time, some delays occur even in the local self-impedance since the delay time influences the integral over positions in the finite volume. We observe that the delay local impedances smoothly vary as positions of the cells and the delay time are increased. In contrast, when we use the center-to-center approximation, all the coordinates within the finite volume simultaneously contribute to the local impedance. Furthermore, when we define the delay time in terms of a pulse function, the delay local mutual-impedance of different positions is defined at the same delay time.

**Figure 7:**
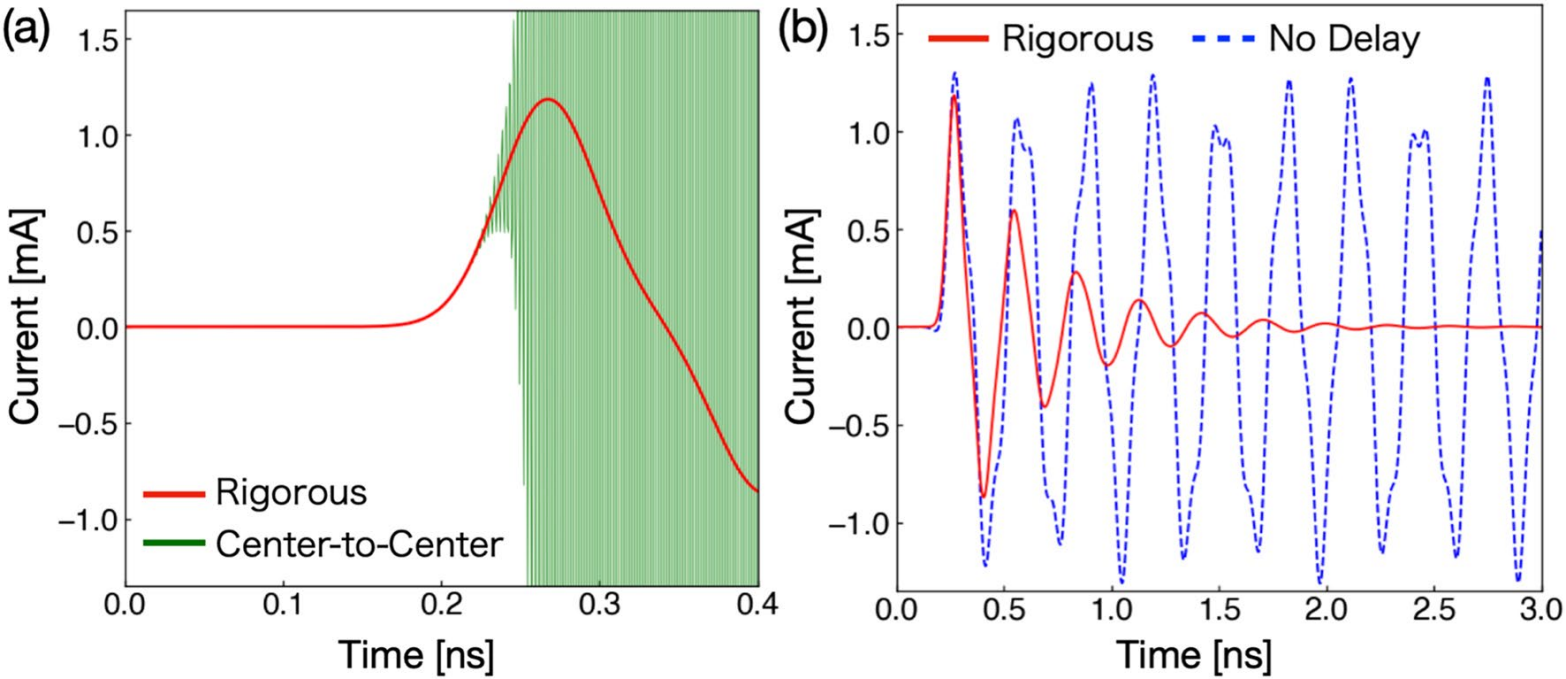
(**a**) and (**b**) are the time variation of the current $I_{1}(t)$ flowing in the center of the single-plane circuit ($x=10$mm), as shown in Fig. 5 with a pulse source current at the boundary. (**a**) compares the two different methods to treat the delay time in the discretization of space and time. (**b**) compares currents in the single-plane circuit in the rigorous method with and without the delay time.

Figure 7(a) compares the numerical results using two different methods in handling the delay time, and (b) compares the numerical results with and without the delay time in the rigorous method. As shown in Fig. 7(a), we see that the numerical results are stable when the delay time is treated rigorously. On the other hand, the calculations using the center-to-center approximation provides unstable results. Therefore, the rigorous treatment of the space-dependent delay time is vital for stable numerical results when we take into account the delay time. We further show numerical results by changing spacial mesh sizes $\Delta x, \Delta y$ and $\alpha$ for the rigorous and approximate cases in Sec. B of Supplementary Information. We find stable solutions in various parameter ranges for the rigorous case, but no stable solutions for the case of the center-to-center approximation in the parameter range explicitly calculated.

Figure 7(b) compares the numerical results with and without the delay time. The current in the single-plane circuit with the delay time is attenuated, caused by the external radiation. On the other hand, when the delay time is not taken into account, the current is not attenuated, and moves back and forth between the two ends of a single-plane antenna. From these results, it is possible to treat the radiation and transmission phenomena from the circuit by taking explicitly the delay time into account. We have made calculations of two plane circuits using the rigorous method together with the center-to-center approximation for the delay term. We have obtained stable solutions in the rigorous method.

## Summary

In this paper, a new numerical calculation method was developed for full-wave analysis in the time domain, considering transmission and radiation in the three-dimensional conductors. The characteristic feature of present numerical method is the simultaneous discretization of time and space using the collocation method, which enables us to treat the delay time rigorously.

We compared numerical results for two cases where we treat the delay time rigorously and approximately using the center-to-center approximation. The results showed that rigorous treatment of the delay time presented in this paper gave a stable numerical result, while the approximate treatment, which uses center-to-center approximation, gave unstable results. For stable numerical calculation of delay time, it was essential to consider the space dependence in the delay term rigorously. We also compared numerical results with and without delay time. The results showed that, in the single-plane circuit, the current significantly decreased in the propagation process due to radiation from the single-plane circuit.

## Supplementary Information

Supplementary Information
