# Supplementary material for: The time domain numerical method of three-dimensional conductors including radiation with lumped parameter circuit
Source: Sci Rep. 2021 Feb 25;11:4598. doi: 10.1038/s41598-021-83916-4 (PMC7907207; doi:10.1038/s41598-021-83916-4)
Supplement: Supplementary file 1 — Supplementary Information [file 41598_2021_83916_MOESM1_ESM.pdf]

Supplementary Information to The time domain numerical method of three-dimensional conductors including radiation with lumped-parameter circuit

Souma Jinno[1], Shuji Kitora[2], Hiroshi Toki[3], and Masayuki Abe[4]

Graduate School of Engineering Science, Osaka University, Toyonaka, Osaka 560-8531, Japan

Abstract

This is a supplemental paper to the paper entitled "The time domain numerical method of three-dimensional conductors including radiation with lumped parameter circuit". We write here the details of derivation of the delay local impedance to be used for the rigorous treatment of the delay terms for stable numerical solutions. We write also the parameter dependence of numerical calculations in detail.

A. Delay Integral Kernel in Multilayer Plane Conductors

The delay local potential coefficient $P^n_{(j,k,l)(j',k',l')}$ in Eq. (32) and delay local inductance $L^n_{\alpha(j,k,l)(j',k',l')}$ in Eq. (34) in the main manuscript can be written using the following delay integral kernel.

$$K^n_{(j,k,l)(j',k',l')} = \int_{V_{(j',k',l')}} \frac{g^n(t_{(j,k,l)}(x',y',z'))}{\sqrt{(x'-x_j)^2+(y'-y_k)^2+(z'-z_l)^2}} \mathrm{d}x'\mathrm{d}y'\mathrm{d}z' \quad (1)$$

We call K^n the delay integral kernel to be calculated for a three dimensional conductor. Here, we limit ourselves to write the case of a plane system as

[1]e-mail: soumajinno117@s.ee.es.osaka-u.ac.jp
[2]e-mail: u760406f@ecs.osaka-u.ac.jp
[3]e-mail: toki@rcnp.osaka-u.ac.jp
[4]e-mail: abe@stec.es.osaka-u.ac.jp

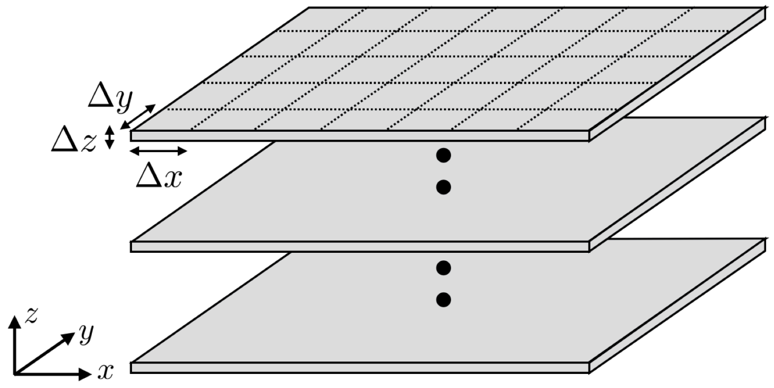

Figure 1: Multiple plane conductors, where the thickness of the plane is denoted as Δz, which is negligibly small. Δx and Δy are the mesh size in the x and y directions.

shown in Figure 1. We consider a very thin planar conductor, and write explicit formula for a plane case. If the thickness Δz of the plane conductor is negligibly small compared to Δx and Δy, we can ignore the change in the z direction.

Thus, in a very thin planar conductor kernel, the integral kernel can be reduced to two dimensions.

$$K^n_{(j,k)(j',k')} = \Delta z \int_{S_{(j',k')}} \frac{g^n(t_{(j,k)}(x',y'))}{\sqrt{(x'-x_j)^2+(y'-y_k)^2}} \mathrm{d}x'\mathrm{d}y' \tag{2}$$

Since the delay integral kernel is a relative relationship between the discretized finite area $S_{(j',k')}$ at a collocation point (x_j, y_k), we derive the delay integral kernel taking (j,k) at the origin: (x_1, y_1). In numerical calculations, further the time step is developed, the more distant effects are generated with the delay effect. The distance of the delay contribution is expressed as the product of the number of delay steps n, the speed of propagation in space v, and the small time interval Δt.

$$r_n = (n+1)v\Delta t \quad (n = 0, 1, \cdots, N_d) \tag{3}$$

The area of delay contribution is a circle of radius r_n centered at the origin (x_1, y_1), which is called the delay circle C_n.

We use the circular coordinate system to formulate the delay integral kernel.

$$x' = r' \cos\theta' \tag{4}$$

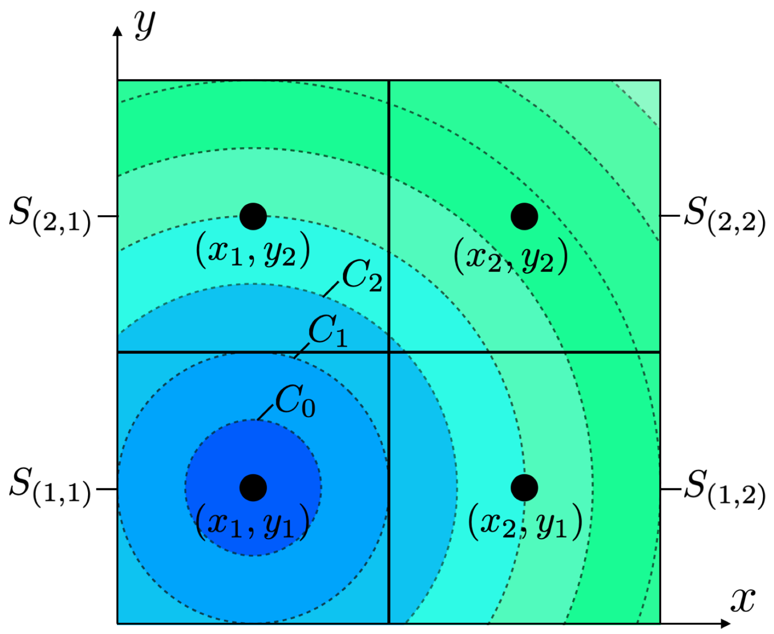

Figure 2: The simplest case for the delay local impedance. Shown here is the case $\Delta x = \Delta y = 4v\Delta t$.

$$y' = r' \sin\theta' \tag{5}$$

The Jacobian in this case is r', which cancels with the denominator. Hence, the delay integral kernel is represented by r' and θ' as,

$$K^n_{(1,1)(j,k)} = \Delta z \int_{S_{(j,k)}} g^n(t_{(1,1)}(r', \theta'))\mathrm{d}r'\mathrm{d}\theta' \ , \tag{6}$$

where $g^n(t_{(1,1)}(r', \theta')) = 1$ within the circle of C_n and outside of the circle of C_{n-1}, as shown in Fig. 2. In other words, the integral range is bounded by the area $S_{(j,k)}$ and the circles C_n and C_{n-1}. The delay integral kernel can be express as follow:

$$K^n_{(1,1)(j,k)} = \Delta z \left(\int_{S_{(j,k)} \cap C_n} \mathrm{d}r'\mathrm{d}\theta' - \int_{S_{(j,k)} \cap C_{n-1}} \mathrm{d}r'\mathrm{d}\theta' \right) \tag{7}$$

To further simplify the calculation, we denote the integral delay kernel derived in the area bounded by the delay circle C_n and the finite area $S_{(j,k)}$

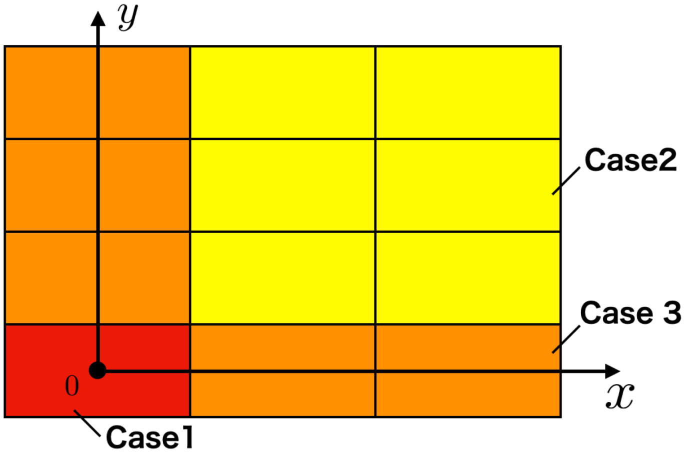

Figure 3: Here shown is the case $\Delta x \neq \Delta y$. There are several cases we have to consider for analytical results.

as $C^n_{(1,1)(j,k)}$ and call it a delay kernel element.

$$C^n_{(1,1)(j,k)} = \int_{S_{(j,k)} \cap C_n} \mathrm{d}r' \mathrm{d}\theta' \tag{8}$$

Therefore, the delay integral kernel to be derived can be expressed using the delay kernel element as follows.

$$K^n_{(1,1)(j,k)} = \Delta z \left(C^n_{(1,1)(j,k)} - C^{n-1}_{(1,1)(j,k)} \right) \tag{9}$$

For general discretization, we consider $\Delta t \geq \Delta x / 2v$and $\Delta y \leq \Delta x$ for a plane conductor case. Here, when we calculate the delay integral kernels, we change the numerical formula depending on the relative position between the origin (x_1, y_1) and the finite area $S_{(j,k)}$ as shown in Fig.3, which are divided by the intersection patterns of $S_{(j,k)}$. We take three cases: Case 1 is the case of delay integral self-kernel, and Case 2 and 3 are delay integral mutual kernels. We are able to obtain analytical expressions for the delay kernels. We use two integral formula:

$$\int d\theta \frac{1}{\cos\theta} = \frac{1}{2} \log \left| \frac{1 + \sin\theta}{1 - \sin\theta} \right| \tag{10}$$

$$\int d\theta \frac{1}{\sin\theta} = \frac{1}{2} \log \left| \frac{1 - \cos\theta}{1 + \cos\theta} \right| \tag{11}$$

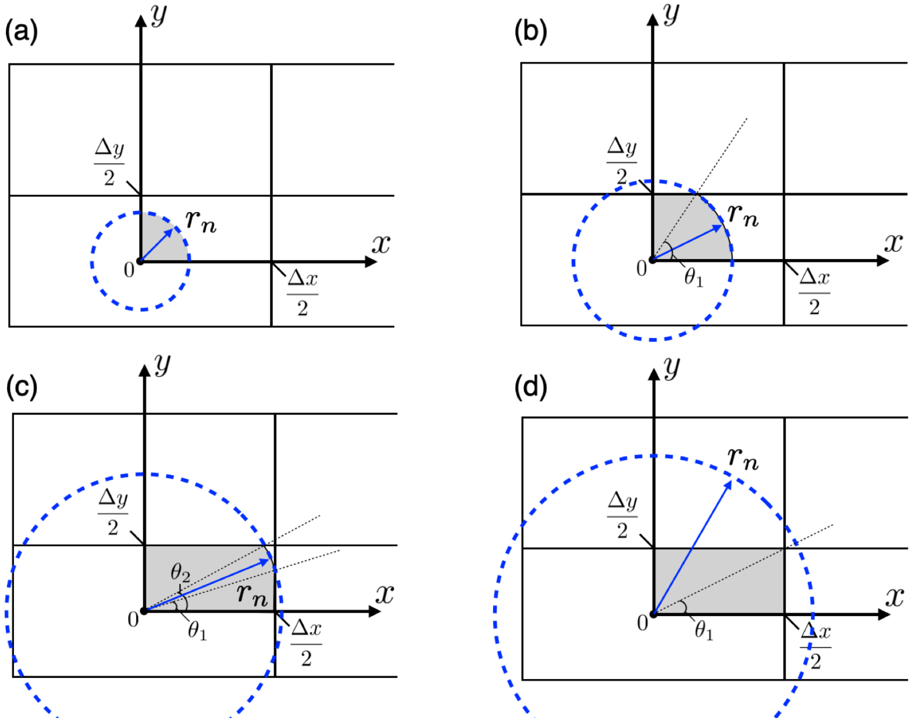

Figure 4: We write 4 cases denoted as (a), (b), (c) and (d). We introduce various angles θ ' s written explicitly in the text.

A.1. Delay self-kernel $(Case1 : j = k = 1)$

When we calculate the delay kernel, the effects within the same finite area are divided by the delay time. Four calculation patterns are there depending on the delay circle C_n and the area $S_{(1,1)}$ shown in Figure 4. We can calculate the delay kernel element in the range of $0 \le \theta' \le \pi/2$ using the delay kernel's symmetry.

A.1.1. (a) : $r_n \le \Delta y/2$

Since the delay circle C_n does not intersect the area $S_{(1,1)}$ as shown in Fig. 4(a), r' does not depend on θ'.

$$C^n_{(0,0)(0,0)} = 2\pi r_n \tag{12}$$

A.1.2. (b) : $\Delta y/2 < r_n \leq \Delta x/2$

The range of r' depends on θ' as shown in Fig. 4(b) where θ_1 is expressed as follow:

$$\theta_1 = \sin^{-1}\left(\frac{\Delta y/2}{r_n}\right) \tag{13}$$

There are contributions from the two areas divided by the θ_1. In one area $0 \leq \theta' \leq \theta_1$, the range of r' is $0 \leq r' \leq r_n$. In the other area $\theta_1 \leq \theta' \leq \frac{\pi}{2}$, the range of r' is $0 \leq r' \leq \Delta y/(2\sin\theta)$. From the above, the delay kernel element is represented as follows:

$$C^n_{(1,1)(1,1)} = 4\theta_1 r_n - \Delta y \log\left|\frac{1-\cos\theta_1}{1+\cos\theta_1}\right| \tag{14}$$

A.1.3. (c) : $\Delta x/2 < r_n \leq \sqrt{(\Delta x/2)^2 + (\Delta y/2)^2}$

The range of r' depends on θ' as shown in Fig. 4 (c). θ_1 and θ_2 are represented as follows:

$$\theta_1 = \cos^{-1}\left(\frac{\Delta x/2}{r_n}\right) \tag{15}$$

$$\theta_2 = \sin^{-1}\left(\frac{\Delta y/2}{r_n}\right) \tag{16}$$

Hence, there are three areas separated by these θ_1 and θ_2. In the first area $0 \leq \theta' \leq \theta_1$, the range of r' is $0 \leq r' \leq \Delta x/(2\cos\theta)$. In the second area $\theta_1 \leq \theta' \leq \theta_2$, the range of r' is $0 \leq r' \leq r_n$. In the third area $\theta_2 \leq \theta' \leq \frac{\pi}{2}$, the range of r' is $0 \leq r' \leq \Delta y/(2\sin\theta)$.

From the above, the delay kernel element is represented as follow:

$$C^n_{(1,1)(1,1)} = \Delta x \log\left|\frac{1+\sin\theta_1}{1-\sin\theta_1}\right| + 4r_n(\theta_2-\theta_1) - \Delta y \log\left|\frac{1-\cos\theta_2}{1+\cos\theta_2}\right| \tag{17}$$

A.1.4. (d) : $\sqrt{(\Delta x/2)^2 + (\Delta y/2)^2} < r_n$

The range of integration of r' depends on θ' as shown in Fig. 4(d), where θ_1 is expressed as follows.

$$\theta_1 = \tan^{-1}\left(\frac{\Delta y}{\Delta x}\right) \tag{18}$$

Hence, there are two areas separated by the θ_1. In the one area $0 \leq \theta' \leq \theta_1$, the range of r' is $0 \leq r' \leq \frac{\Delta x/2}{\cos\theta}$ In the other area $\theta_1 \leq \theta' \leq \frac{\pi}{2}$, the range of r' is $0 \leq r' \leq \frac{\Delta y/2}{\sin\theta}$. From the above, the delay kernel element is represented as follow:

$$C^n_{(1,1)(1,1)} = \Delta x \log\left|\frac{1+\sin\theta_1}{1-\sin\theta_1}\right| - \Delta y \log\left|\frac{1-\cos\theta_1}{1+\cos\theta_1}\right| \tag{19}$$

A.2. Delay mutual kernel $(Case2 : j \neq k \text{ and } 2 \leq j \text{ and } 2 \leq k)$

We define the corner points $x_{j0}, x_{j1}, y_{k0}, y_{k1}$ of the finite area $S_{(j,k)}$ in Fig. 5 as follows:

$$x_{j0} = \Delta x \times \left(j - \frac{3}{2}\right) \tag{20}$$

$$x_{j1} = \Delta x \times \left(j - \frac{1}{2}\right) \tag{21}$$

$$y_{k0} = \Delta y \times \left(k - \frac{3}{2}\right) \tag{22}$$

$$y_{k1} = \Delta y \times \left(k - \frac{1}{2}\right) \tag{23}$$

Here, there are five cases regarding C^n and $S_{(j,k)}$ in Fig. 5, from (a) to (e), and the formulation of the delay kernel element varies in each case.

A.2.1. (a) : $\sqrt{x_{j0}^2 + y_{k0}^2} \leq r_n \leq \sqrt{x_{j0}^2 + y_{k1}^2}$

The range of r' depends on θ' as shown in Fig. 5(a), θ_1, θ_2 and θ_3 are expressed as follows:

$$\theta_1 = \sin^{-1}\left(\frac{y_{k0}}{r_n}\right) \tag{24}$$

$$\theta_2 = \tan^{-1}\left(\frac{y_{k0}}{x_{j0}}\right) \tag{25}$$

$$\theta_3 = \cos^{-1}\left(\frac{x_{j0}}{r_n}\right) \tag{26}$$

There are two areas contributing to the delay kernel. In one area $\theta_1 \leq \theta' \leq \theta_2$, the range of r' is $\frac{y_{k0}}{\sin\theta'} (= r_1) \leq r' \leq r_n$. In the second area $\theta_2 \leq \theta' \leq \theta_3$,

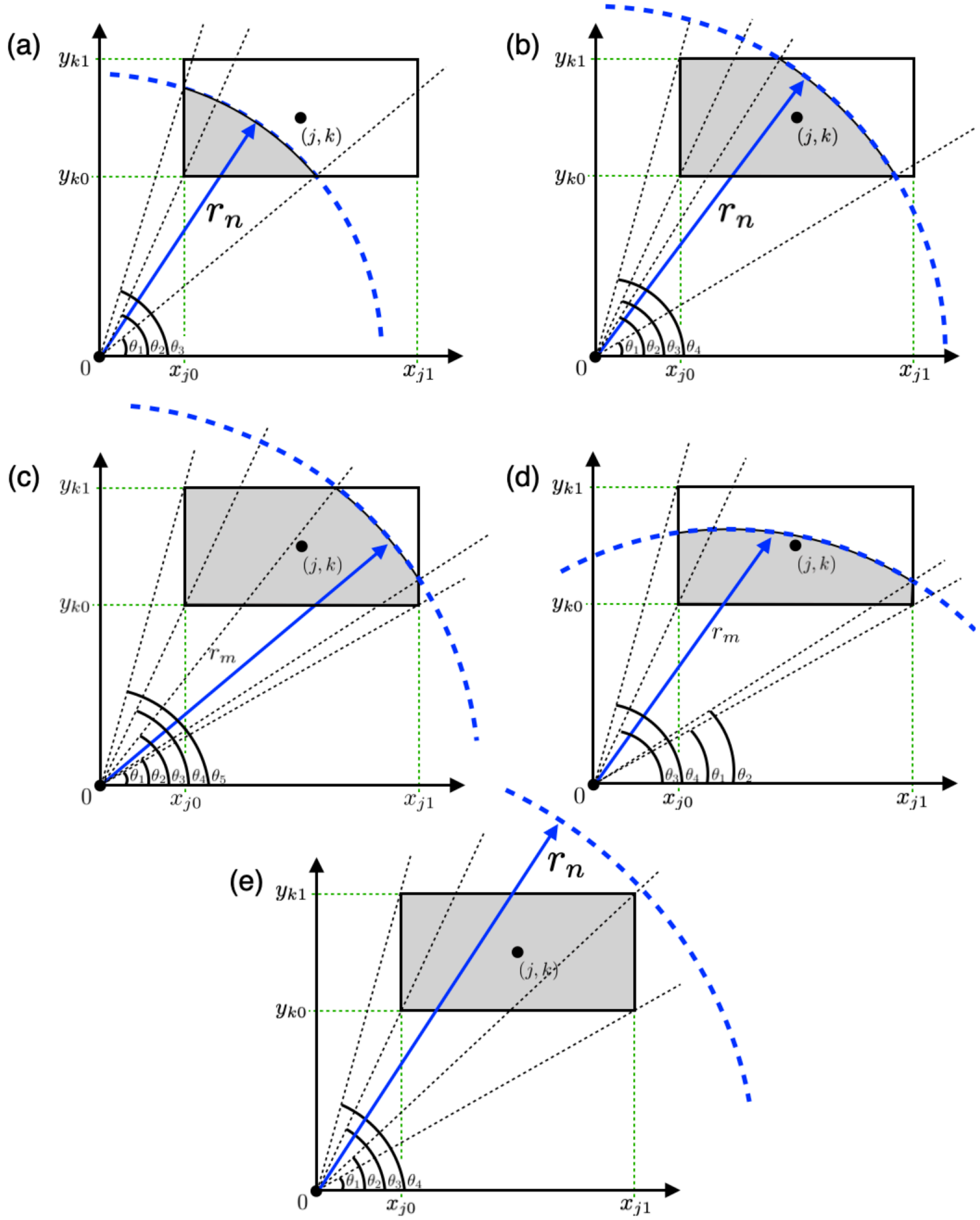

Figure 5: There are five cases and various quantities as θ ' s are expressed in the text.

the range of r' is $\frac{x_{j0}}{\cos\theta'}\,(=r_2) \leq r' \leq r_n$. From the above, the delay kernel

element is represented as follow:

$$C^n_{(1,1)(j,k)} = (\theta_3 - \theta_1) r_n - \frac{y_{k0}}{2} \left[\log \left| \frac{1-\cos\theta'}{1+\cos\theta'} \right| \right]_{\theta_1}^{\theta_2} - \frac{x_{j0}}{2} \left[\log \left| \frac{1+\sin\theta'}{1-\sin\theta'} \right| \right]_{\theta_2}^{\theta_3} \tag{27}$$

A.2.2. (b) : $\sqrt{x_{j0}^2 + y_{k1}^2} \leq \sqrt{x_{j1}^2 + y_{k0}^2}$ *and* $\sqrt{x_{j0}^2 + y_{k1}^2} \leq r_n \leq \sqrt{x_{j1}^2 + y_{k0}^2}$

The range of r' depends on θ' as shown in Fig. 5(b), $\theta_1, \theta_2, \theta_3$ and θ_4 are expressed as follows:

$$\theta_1 = \sin^{-1}\left(\frac{y_{k0}}{r_n}\right) \tag{28}$$

$$\theta_2 = \sin^{-1}\left(\frac{y_{k1}}{r_n}\right) \tag{29}$$

$$\theta_3 = \tan^{-1}\left(\frac{y_{k0}}{x_{j0}}\right) \tag{30}$$

$$\theta_4 = \tan^{-1}\left(\frac{y_{k1}}{x_{j0}}\right) \tag{31}$$

There are three areas contributing to the delay kernel. In the first area $\theta_1 \leq \theta' \leq \theta_2$, the range of r' is $\frac{y_{k0}}{\sin\theta'} (= r_1) \leq r' \leq r_n$. In the second area $\theta_2 \leq \theta' \leq \theta_3$, the range of r' is $\frac{y_{k0}}{\sin\theta'} (= r_1) \leq r' \leq \frac{y_{k1}}{\sin\theta'} (= r_2)$. In the third area $\theta_3 \leq \theta' \leq \theta_4$, the range of r' is $\frac{x_{j0}}{\cos\theta'} (= r_3) \leq r' \leq \frac{y_{k1}}{\sin\theta'} (= r_2)$. From the above, the delay kernel element is represented as follow:

$$\begin{aligned} C^n_{(1,1)(j,k)} &= (\theta_2 - \theta_1) r_n - \frac{y_{k0}}{2} \left[\log \left| \frac{1-\cos\theta'}{1+\cos\theta'} \right| \right]_{\theta_1}^{\theta_3} \\ &\quad + \frac{y_{k1}}{2} \left[\log \left| \frac{1-\cos\theta'}{1+\cos\theta'} \right| \right]_{\theta_2}^{\theta_4} - \frac{x_{j0}}{2} \left[\log \left| \frac{1+\sin\theta'}{1-\sin\theta'} \right| \right]_{\theta_3}^{\theta_4} \end{aligned} \tag{32}$$

A.2.3. (c) : $\sqrt{x_{j1}^2 + y_{k0}^2} \leq \sqrt{x_{j0}^2 + y_{k1}^2}$ *and* $\sqrt{x_{j1}^2 + y_{k0}^2} \leq r_n \leq \sqrt{x_{j0}^2 + y_{k1}^2}$

The range of r' depends on θ' as shown in Fig. 5(c), $\theta_1, \theta_2, \theta_3$ and θ_4 are expressed as follows:

$$\theta_1 = \tan^{-1}\left(\frac{y_{k0}}{x_{j1}}\right) \tag{33}$$

$$\theta_2 = \cos^{-1}\left(\frac{x_{j1}}{r_n}\right) \tag{34}$$

$$\theta_3 = \tan^{-1}\left(\frac{y_{k0}}{x_{j0}}\right) \tag{35}$$

$$\theta_4 = \cos^{-1}\left(\frac{x_{j0}}{r_n}\right) \tag{36}$$

There are three areas contributing to the delay kernel. In the first area $\theta_1 \le \theta' \le \theta_2$, the range of r' is $\frac{y_{k0}}{\sin\theta'} (= r_1(\theta')) \le r' \le \frac{x_{j1}}{\cos\theta'} (= r_2(\theta'))$. In the second area $\theta_2 \le \theta' \le \theta_3$, the range of r' is $\frac{y_{k0}}{\sin\theta'} (= r_1(\theta')) \le r' \le r_n$. In the third area $\theta_3 \le \theta' \le \theta_4$, the range of r' is $\frac{x_{j0}}{\cos\theta'} (= r_3(\theta')) \le r' \le r_n$. From the above, the delay kernel element is represented as follow:

$$\begin{aligned} C^n_{(1,1)(j,k)} &= \frac{x_{j1}}{2}\left[\log\left|\frac{1+\sin\theta'}{1-\sin\theta'}\right|\right]_{\theta_1}^{\theta_2} - \frac{y_{k0}}{2}\left[\log\left|\frac{1-\cos\theta'}{1+\cos\theta'}\right|\right]_{\theta_1}^{\theta_3} \\ &\quad +(\theta_4-\theta_2)r_n - \frac{x_{j0}}{2}\left[\log\left|\frac{1+\sin\theta'}{1-\sin\theta'}\right|\right]_{\theta_3}^{\theta_4} \end{aligned} \tag{37}$$

A.2.4. (d) : $\sqrt{x_{j1}^2 + y_{k0}^2} \le r_n \le \sqrt{x_{j1}^2 + y_{k1}^2}$

The range of r' depends on θ' as shown in Fig. 5(d), $\theta_1, \theta_2, \theta_3, \theta_4$ and θ_5 are expressed as follows:

$$\theta_1 = \tan^{-1}\left(\frac{y_{k0}}{x_{j1}}\right) \tag{38}$$

$$\theta_2 = \cos^{-1}\left(\frac{x_{j1}}{r_n}\right) \tag{39}$$

$$\theta_3 = \sin^{-1}\left(\frac{y_{k1}}{r_n}\right) \tag{40}$$

$$\theta_4 = \tan^{-1}\left(\frac{y_{k0}}{x_{j0}}\right) \tag{41}$$

$$\theta_5 = \tan^{-1}\left(\frac{y_{k1}}{x_{j0}}\right) \tag{42}$$

There are four areas contributing to the delay kernel. In the first area $\theta_1 \le \theta' \le \theta_2$, the range of r' is $\frac{y_{k0}}{\sin\theta'} (= r_1(\theta')) \le r' \le \frac{x_{j1}}{\cos\theta'} (= r_2(\theta'))$. In the second area $\theta_2 \le \theta' \le \theta_3$, the range of r' is $\frac{y_{k0}}{\sin\theta'} (= r_1(\theta')) \le r' \le r_n$. In the third

area $\theta_3 \leq \theta' \leq \theta_4$, the range of r' is $\frac{y_{k0}}{\sin\theta'}\,(=r_1(\theta')) \leq r' \leq \frac{y_{k1}}{\sin\theta'}\,(=r_3(\theta'))$. In the fourth area $\theta_4 \leq \theta' \leq \theta_5$, the range of r' is $\frac{x_{j0}}{\cos\theta'}\,(=r_4(\theta')) \leq r' \leq \frac{y_{k1}}{\sin\theta'}\,(=r_3(\theta'))$. From the above, the delay kernel element is represented as follow:

$$\begin{aligned} C^n_{(1,1)(j,k)} &= \frac{x_{j1}}{2}\left[\log\left|\frac{1+\sin\theta'}{1-\sin\theta'}\right|\right]_{\theta_1}^{\theta_2} - \frac{y_{k0}}{2}\left[\log\left|\frac{1-\cos\theta'}{1+\cos\theta'}\right|\right]_{\theta_1}^{\theta_4} + (\theta_3-\theta_2)r_n \\ &\quad + \frac{y_{k1}}{2}\left[\log\left|\frac{1-\cos\theta'}{1+\cos\theta'}\right|\right]_{\theta_3}^{\theta_5} - \frac{x_{j0}}{2}\left[\log\left|\frac{1+\sin\theta'}{1-\sin\theta'}\right|\right]_{\theta_4}^{\theta_5} \end{aligned} \tag{43}$$

A.2.5. (e) : $\sqrt{x_{j1}^2+y_{k1}^2} \leq r_n$

The range of r' depends on θ' as shown in Fig. 5(d), $\theta_1, \theta_2, \theta_3$ and θ_4 are expressed as follows:

$$\theta_1 = \tan^{-1}\left(\frac{y_{k0}}{x_{j1}}\right) \tag{44}$$

$$\theta_2 = \tan^{-1}\left(\frac{y_{k1}}{x_{j1}}\right) \tag{45}$$

$$\theta_3 = \tan^{-1}\left(\frac{y_{k0}}{x_{j0}}\right) \tag{46}$$

$$\theta_4 = \tan^{-1}\left(\frac{y_{k1}}{x_{j0}}\right) \tag{47}$$

There are three areas contributing to the delay kernel. In the first area $\theta_1 \leq \theta' \leq \theta_2$, the range of r' is $\frac{y_{k0}}{\sin\theta'}\,(=r_1(\theta')) \leq r' \leq \frac{x_{j1}}{\cos\theta'}\,(=r_2(\theta'))$. In the second area $\theta_2 \leq \theta' \leq \theta_3$, the range of r' is $\frac{y_{k0}}{\sin\theta'}\,(=r_1(\theta')) \leq r' \leq \frac{y_{k1}}{\sin\theta'}\,(=r_3(\theta'))$. In the third area $\theta_3 \leq \theta' \leq \theta_4$, the range of r' is $\frac{x_{j0}}{\cos\theta'}\,(=r_4(\theta')) \leq r' \leq \frac{y_{k1}}{\sin\theta'}\,(=r_3(\theta'))$. From the above, the delay kernel element is represented as follows:

$$\begin{aligned} C^n_{(1,1)(j,k)} &= \frac{x_{j1}}{2}\left[\log\left|\frac{1+\sin\theta'}{1-\sin\theta'}\right|\right]_{\theta_1}^{\theta_2} - \frac{y_{k0}}{2}\left[\log\left|\frac{1-\cos\theta'}{1+\cos\theta'}\right|\right]_{\theta_1}^{\theta_3} \\ &\quad + \frac{y_{k1}}{2}\left[\log\left|\frac{1-\cos\theta'}{1+\cos\theta'}\right|\right]_{\theta_2}^{\theta_4} - \frac{x_{j0}}{2}\left[\log\left|\frac{1+\sin\theta'}{1-\sin\theta'}\right|\right]_{\theta_3}^{\theta_4} \end{aligned} \tag{48}$$

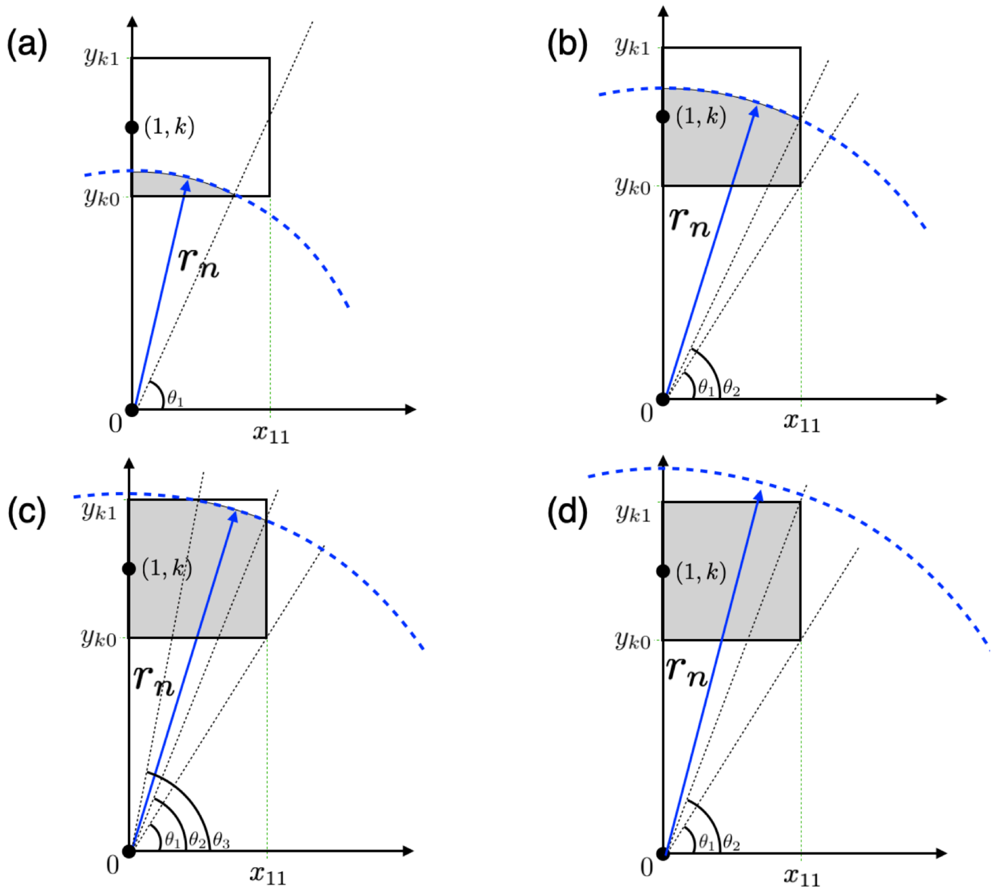

Figure 6: There are 4 cases in this geometry. We write quantities as θ ' s in the text explicitly.s

A.3. Delay mutual kernel (*Case3* : $j = 1$ *and* $k \geq 2$ *or* $k = 1$ *and* $j \geq 2$)

We define the corners points x_{11}, y_{k0} and y_{k1} of the finite area $S_{(j,k)}$ in Fig. 5, which is the case of $j = 1$ and $k \geq 2$, as follows:

$$x_{11} = \frac{\Delta x}{2} \tag{49}$$

$$y_{k0} = \Delta y \times \left(k - \frac{3}{2}\right) \tag{50}$$

$$y_{k1} = \Delta y \times \left(k - \frac{1}{2}\right) \tag{51}$$

When $S_{(j,k)}$ exists on the x-axis, which is the case of $k = 1$ and $j \geq 2$, we can swap the relationship between x and y in the delay kernel.

A.3.1. (a) : $y_{k0} \leq r_n < \sqrt{x_{11}^2 + y_{k0}^2}$

The range of r' depends on θ' as shown in Fig. 6(a), θ_1 is expressed as follow:

$$\theta_1 = \sin^{-1}\left(\frac{y_{k0}}{r_n}\right) \tag{52}$$

In the small area $\theta_1 \leq \theta' \leq \frac{\pi}{2}$, the range of r' is $\frac{y_{k0}}{\sin\theta'}\,(= r_1) \leq r' \leq r_n$. Therefore, the delay kernel element is represented as follow:

$$C^n_{(1,1)(j,k)} = (\pi - 2\theta_1)\, r_n + y_{k0} \log\left|\frac{1-\cos\theta_1}{1+\cos\theta_1}\right| \tag{53}$$

A.3.2. (b) : $\sqrt{x_{11}^2 + y_{k0}^2} \leq r_n < y_{k1}$

The range of r' depends on θ' as shown in Fig. 6(b), θ_1 and θ_2 are expressed as follows:

$$\theta_1 = \tan^{-1}\left(\frac{y_{k0}}{x_{11}}\right) \tag{54}$$

$$\theta_2 = \cos^{-1}\left(\frac{x_{11}}{r_n}\right) \tag{55}$$

We have two areas contributing to the delay kernel. In the one area $\theta_1 \leq \theta' \leq \theta_2$, the range of r' is $\frac{y_{k0}}{\sin\theta'}\,(= r_1(\theta')) \leq r' \leq \frac{x_{11}}{\cos\theta'}\,(= r_2(\theta'))$. In the other area $\theta_2 \leq \theta' \leq \frac{\pi}{2}$, the range of r' is $\frac{y_{k0}}{\sin\theta'}\,(= r_1(\theta')) \leq r' \leq r_n$. From the above, the delay kernel element is represented as follows:

$$C^n_{(1,1)(j,k)} = x_{11}\left[\log\left|\frac{1+\sin\theta'}{1-\sin\theta'}\right|\right]_{\theta_1}^{\theta_2} - y_{k0}\left[\log\left|\frac{1-\cos\theta'}{1+\cos\theta'}\right|\right]_{\theta_1}^{\frac{\pi}{2}} + (\pi - 2\theta_2)\, r_n \tag{56}$$

A.3.3. (c) : $y_{k1} \leq r_n < \sqrt{x_{11}^2 + y_{k1}^2}$

The range of r' depends on θ' as shown in Fig. 6(c), θ_1, θ_2 and θ_3 are expressed as follows:

$$\theta_1 = \tan^{-1}\left(\frac{y_{k0}}{x_{j1}}\right) \tag{57}$$

$$\theta_2 = \cos^{-1}\left(\frac{x_{11}}{r_n}\right) \tag{58}$$

$$\theta_3 = \sin^{-1}\left(\frac{y_{k1}}{r_n}\right) \tag{59}$$

We have three areas contributing to the delay kernel. In the first area $\theta_1 \leq \theta' \leq \theta_2$, the range of r' is $\frac{y_{k0}}{\sin\theta'}\,(= r_1(\theta')) \leq r' \leq \frac{x_{11}}{\cos\theta'}\,(= r_2(\theta'))$. In the second area $\theta_2 \leq \theta' \leq \theta_3$, the range of r' is $\frac{y_{k0}}{\sin\theta'}\,(= r_1(\theta')) \leq r' \leq r_n$. In the third area $\theta_3 \leq \theta' \leq \frac{\pi}{2}$, the range of r' is $\frac{y_{k0}}{\sin\theta'}\,(= r_1(\theta')) \leq r' \leq \frac{y_{k1}}{\sin\theta'}\,(= r_3(\theta'))$. From the above, the delay kernel element is represented as follows:

$$\begin{aligned} C^n_{(1,1)(j,k)} &= x_{11}\left[\log\left|\frac{1+\sin\theta'}{1-\sin\theta'}\right|\right]_{\theta_1}^{\theta_2} - y_{k0}\left[\log\left|\frac{1-\cos\theta'}{1+\cos\theta'}\right|\right]_{\theta_1}^{\frac{\pi}{2}} \\ &\quad +2\left(\theta_3-\theta_2\right) r_n + y_{k1}\left[\log\left|\frac{1-\cos\theta'}{1+\cos\theta'}\right|\right]_{\theta_3}^{\frac{\pi}{2}} \end{aligned} \tag{60}$$

A.3.4. (d) : $\sqrt{x_{11}^2 + y_{k1}^2} \leq r_n$

The range of r' depends on θ' as shown in Fig. 6(d), θ_1 and θ_2 are expressed as follows:

$$\theta_1 = \tan^{-1}\left(\frac{y_{k0}}{x_{11}}\right) \tag{61}$$

$$\theta_2 = \tan^{-1}\left(\frac{y_{k1}}{x_{11}}\right) \tag{62}$$

There are two areas contributing to the delay kernel. In the one area $\theta_1 \leq \theta' \leq \theta_2$, the range of r' is $\frac{y_{k0}}{\sin\theta'}\,(= r_1(\theta')) \leq r' \leq \frac{x_{11}}{\cos\theta'}\,(= r_2(\theta'))$. In the second area $\theta_2 \leq \theta' \leq \frac{\pi}{2}$, the range of r' is $\frac{y_{k0}}{\sin\theta'}\,(= r_1(\theta')) \leq r' \leq \frac{y_{k1}}{\sin\theta'}\,(= r_3(\theta'))$. From the above, the delay kernel element is represented as follows:

$$\begin{aligned} C^n_{(1,1)(j,k)} &= x_{11}\left[\log\left|\frac{1+\sin\theta'}{1-\sin\theta'}\right|\right]_{\theta_1}^{\theta_2} - y_{k0}\left[\log\left|\frac{1-\cos\theta'}{1+\cos\theta'}\right|\right]_{\theta_1}^{\frac{\pi}{2}} \\ &\quad +y_{k1}\left[\log\left|\frac{1-\cos\theta'}{1+\cos\theta'}\right|\right]_{\theta_2}^{\frac{\pi}{2}} \end{aligned} \tag{63}$$

Until here all the formula are developed to express the delay kernels analytically to be used for rigorous calculations for thin plane conductors. We are able to write the delay kernels for the case of multi-layer conductors in the

similar manner. In principle we are able to write all the necessary formula analytically for all the cases of three dimensional conductors. However, it may be better to use numerical integrations for the delay kernels for a small local volume with the condition of the time pulse function in the delay term. In this case, the analytical expressions shown here can be used for the check of numerical calculations.

B. Numerical consideration on stability and convergence

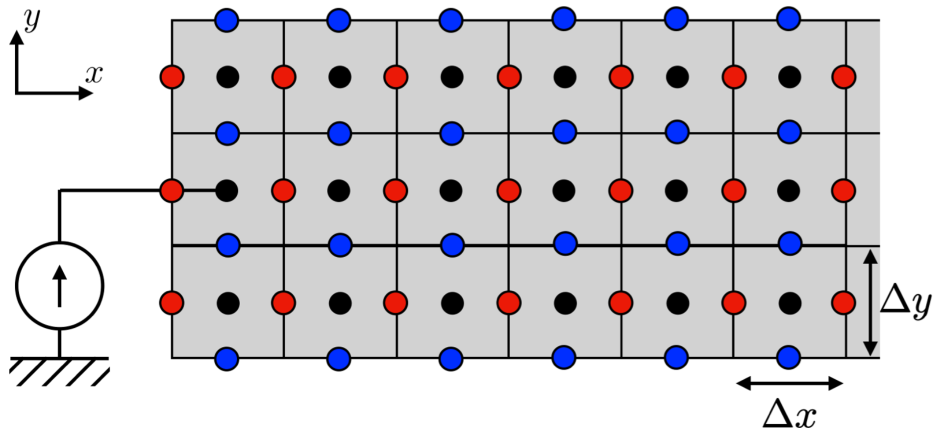

Figure 7: The areas separated by various meshes in the x- and y-directions and collocation points in the spacial discretization of the circuit conductor. The mesh lines represented by the solid line define the finite area of potential with the size Δx and Δy. The black points at the centers of finite areas are collocation points of the potential, the red points at a half-integer shifted in the x-direction from the potential points are collocation points of the current density flowing in the x-direction, and the blue points at a half-integer shifted in the y-direction are collocation points of the current density flowing in the y-direction.

This section shows the stability and convergence of the results of proposed numerical method. The time-domain integral-differential equation including delay time suffers from the "late-time instability" problems. In this section, we discuss various cases in the parameter space when the late-time instability does not occur based on numerical results.

We use the same circuit configuration of Fig. 5 in the main manuscript. Figure 7 shows areas separated by various meshes in the x- and y-directions and collocation points for potential and current density in the left hand side

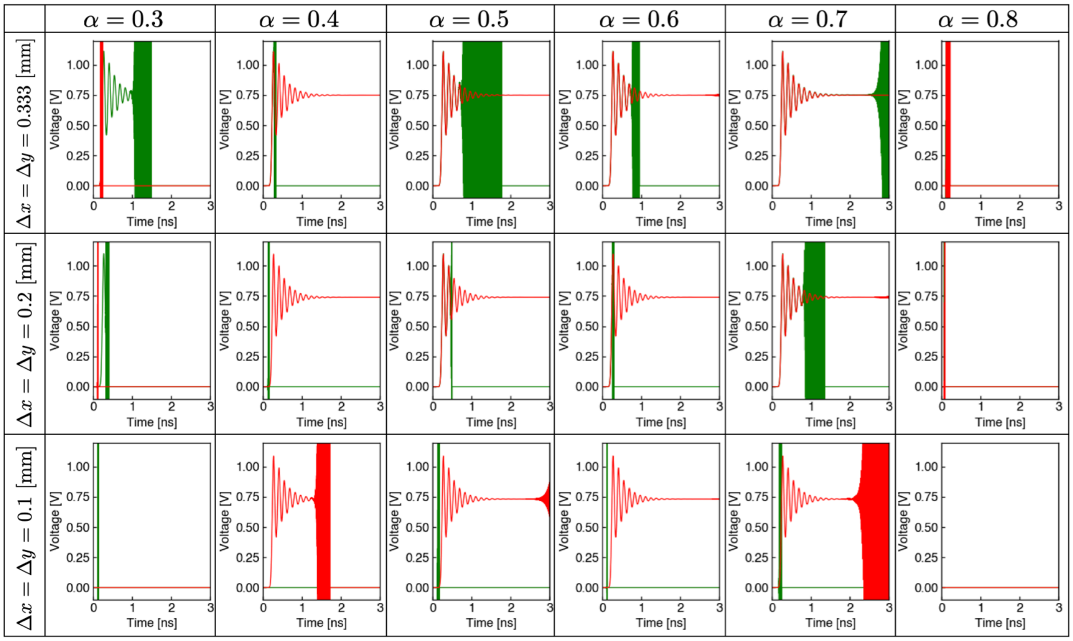

Figure 8: This table shows numerical results of the potential at the center($x = 0.5$ m) of the circuit shown in Fig. 5 of main manuscript as a function of time. The red lines are the case of the proposed method and green lines are the cease of the center-to-center approximation. The size of discretized space and time are changed to consider the stability. In the rows, potentials are shown with the same size of discretized space $\Delta x, \Delta y$ for various $\alpha(= v\Delta x/\Delta t)$. In the columns, potentials are shown with the same α for various $\Delta x, \Delta y$.

of the conductor with the input current. We input a Gaussian function in the left hand side of the conductor, and calculate the potential and current density taking various parameter sets. Figure 8 shows the numerical results calculated taking various Δx keeping $\Delta y = \Delta x$ and various $\alpha(= v\Delta t/\Delta x)$, which determines Δt.

The results calculated by the proposed method, colored by red, show various stable regions and the cases with late time instability. The results of $\Delta x = \Delta y = 0.333$ and $\Delta x = \Delta y = 0.2$ are stable between $\alpha = 0.4$ and 0.7. The results of $\Delta x = \Delta y = 0.1$ are stable around $\alpha = 0.5, 0.6$, but show the late time instability for $\alpha = 0.3, 0.7$. For the cases which is smaller than $\alpha = 0.3$ and larger than $\alpha = 0.8$, we do not find stable solutions. We do not yet have a precise mathematical explanation of these stability behaviors. We

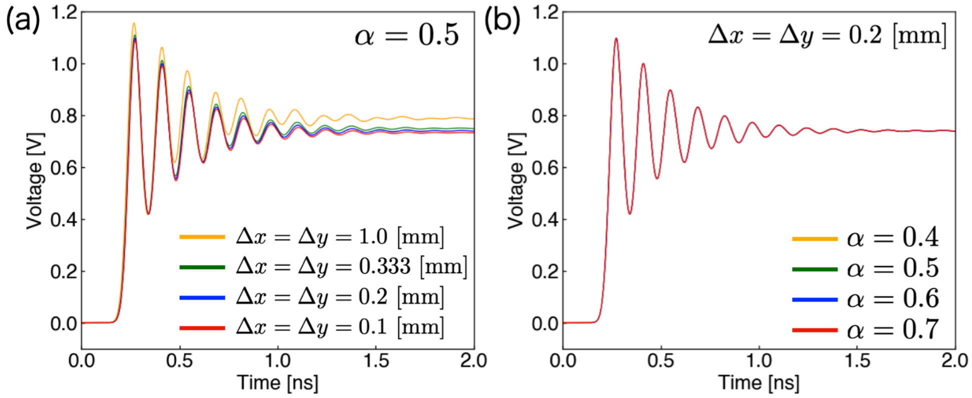

Figure 9: (a) Numerical results of potential at the middle of the conductor as functions of time for various Δx and Δy with 1.0 mm (orange line), 0.333 mm (green line), 0.2 mm (blue line), and 0.1 mm (red line), where α is fixed to 0.5. (b) Numerical results for various α with 0.4 (orange line), 0.5 (green line), 0.6 (blue line) and 0.7 (red line), where Δx and Δy is fixed to 0.2 mm.

perform similar calculations with the center-to-center approximation, which is colored by green in Fig. 8. We do not get stable results in the parameter range shown in this figure.

Furthermore, we show the results calculated by the proposed method in Fig. 9, where the sizes of discretized space and time are changed. Figure 9 (a) shows the convergence regarding the size of discretized space Δx and Δy while keeping $\alpha = 0.5$. From the results, the numerical results converge as the size of the discretized space gets small. The result of $\Delta x = \Delta y = 1$ mm deviates from other cases. However, when the mesh size is reduced to a smaller size, the results converge quickly. Figure 9 (b) shows the convergence regarding the size of discretized time, while keeping $\Delta x = \Delta y = 0.2$ mm. This result shows that the α dependence is negligibly small once the numerical results are stable.
